# The Decidability of Verification under PS 2.0

**DOI:** 10.1007/978-3-030-72019-3_1

**Published:** 2021-03-23

**Authors:** Parosh Aziz Abdulla, Mohamed Faouzi Atig, Adwait Godbole, S. Krishna, Viktor Vafeiadis

**Affiliations:** Imperial College, London, UK; 9 Uppsala University, Uppsala, Sweden; 10 IIT Bombay, Mumbai, India; 11 MPI-SWS, Kaiserslautern, Germany

**Keywords:** Model-Checking, Memory Models, Promising Semantics

## Abstract

We consider the reachability problem for finite-state multi-threaded programs under the *promising semantics* (PS 2.0) of Lee et al., which captures most common program transformations. Since reachability is already known to be undecidable in the fragment of PS 2.0 with only release-acquire accesses (PS 2.0-ra), we consider the fragment with only relaxed accesses and promises (PS 2.0-rlx). We show that reachability under PS 2.0-rlx is undecidable in general and that it becomes decidable, albeit non-primitive recursive, if we bound the number of promises.

Given these results, we consider a bounded version of the reachability problem. To this end, we bound both the number of promises and of “*view-switches*”, i.e., the number of times the processes may switch their local views of the global memory. We provide a code-to-code translation from an input program under PS 2.0 (with relaxed and release-acquire memory accesses along with promises) to a program under SC, thereby reducing the bounded reachability problem under PS 2.0 to the bounded context-switching problem under SC. We have implemented a tool and tested it on a set of benchmarks, demonstrating that typical bugs in programs can be found with a small bound.

## 1 Introduction

An important long-standing open problem in PL research has been to define a weak memory model that captures the semantics of concurrent memory accesses in languages like Java and C/C++. A model is considered good if it can be implemented efficiently (i.e., if it supports all usual compiler optimizations and its accesses are compiled to plain x86/ARM/Power/RISCV accesses), and is easy to reason about. To address this problem, Kang et al. [16] introduced the *promising semantics*. This was the first model that supported basic invariant reasoning, the DRF guarantee, and even a non-trivial program logic [30].

In the promising semantics, the memory is modeled as a set of timestamped messages, each corresponding to a write made by the program. Each process/thread records its own view of the memory—i.e., the latest timestamp for each memory location that it is aware of. A message has the form $(x, v, (f, t], V)$ where $x$ is a location, $v$ a value to be stored for $x$, $(f, t]$ is the timestamp interval corresponding to the write and $V$ is the local view of the process who made the write to $x$. When reading from memory, a process can either return the value stored at the timestamp in its view or advance its view to some larger timestamp and read from that message. When a process $p$ writes to memory location $x$, a new message with a timestamp larger than $p$'s view of $x$ is created, and $p$'s view is advanced to include the new message. In addition, in order to allow load-store reorderings, a process is allowed to *promise* a certain write in the future. A promise is also added as a message in the memory, except that the local view of the process is not updated using the timestamp interval in the message. This is done only when the promise is eventually fulfilled. A *consistency* check is used to ensure that every promised message can be *certified* (i.e., made fulfillable) by executing that process on its own. Furthermore, this should hold from any future memory (i.e., from any extension of the memory with additional messages). The quantification prevents deadlocks (i.e., processes from making promises they are not able to fulfil). However, the unbounded number of future memories, that need to be checked, makes the verification of even simple programs practically infeasible. Moreover, a number of transformations based on global value range analysis as well as register promotion were not supported in [16].

To address these concerns, Lee et al. developed a new version of the promising semantics, PS 2.0 [22] PS 2.0 simplifies the consistency check and instead of checking the promise fulfilment from all future memories, PS 2.0 checks for promise fulfilment only from a specially crafted extension of the current memory called capped memory. PS 2.0 also introduces the notion of reservations, which allows a process to secure a timestamp interval in order to perform a future atomic read-modify-write instruction. The reservation blocks any other message from using that timestamp interval. Because of these changes, PS 2.0 supports register promotion and global value range analysis, while capturing all features (process local optimizations, DRF guarantees, hardware mappings) of the original promising semantics. Although PS 2.0 can be considered a semantic breakthough, it is a very complex model: it supports two memory access modes, relaxed (`rlx`) and release-acquire (`ra`), along with promises, reservations and certifications.

Let PS 2.0-rlx (resp. PS 2.0-`ra`) be the fragment of PS 2.0 allowing only relaxed (`rlx`) (resp. release-acquire (`ra`)) memory accesses. A natural and fundamental question to investigate is the verification of concurrent programs under PS 2.0. Consider the reachability problem, i.e., whether a given configuration of a concurrent finite-state program is reachable. Reachability with only `ra` accesses has already been shown to be undecidable [1], even without promises and reservations. That leaves us only the PS 2.0-rlx fragment, which captures the semantics of concurrent 'relaxed' memory accesses in programming languages such as Java and C/C++. We show that if an unbounded number of promises is allowed, the reachability problem under PS 2.0-rlx is undecidable. Undecidability is obtained with an execution with only 2 processes and 3 context switches, where a context is a computation segment in which only one process is active.

Then, we show that reachability under PS 2.0-rlx becomes decidable if we bound the number of promises at any time (however, the total number of promises made within a run can be unbounded). The proof introduces a new memory model with higher order words LoHoW, which we show equivalent to PS 2.0-rlx in terms of reachable states. Under the bounded promises assumption, we use the decidability of the coverability problem of well structured transition systems (WSTS) [7,13] to show that the reachability problem for LoHoW with bounded number of promises is decidable. Further, PS 2.0-rlx without promises and reservations has a non-primitive recursive lower bound. Our decidability result covers the relaxed fragment of the RC11 model [20,16] (which matches the PS 2.0-rlx fragment with no promises). Given the high complexity for PS 2.0-rlx and the undecidability of PS 2.0-ra, we next consider a bounded version of the reachability problem. To this end, we propose a parametric under-approximation in the spirit of *context bounding* [9,33,21,26,24,29,1,3]. The aim of context bounding is to restrict the otherwise unbounded interaction between processes, and has been shown experimentally in the case of SC programs to maintain enough behaviour coverage for bug detection [24,29]. The concept of context bounding has been extended for weak memory models. For instance, for RA, Abdula et al. [1] proposed *view bounding* using the notion of view-switching messages and a translation that keeps track of the causality between different variables. Since PS 2.0 subsumes RA, we propose a bounding notion that extends view bounding.

Using our new bounding notion, we propose a source-to-source translation from programs under PS 2.0 to context-bounded executions of the transformed program under SC. The challenges in our translation differ a lot from that in [1], as we have to provide a procedure that (i) handles different memory accesses `rlx` and `ra`, (ii) guesses the promises and reservations in a non-deterministic manner, and (iii) verifies that promises are fulfilled using the capped memory.

We have implemented this reduction in a tool, PS2SC. Our experimental results demonstrate the effectiveness of our approach. We exhibit cases where hard-to-find bugs are detectable using a small view-bound. Our tool displays resilience to trivial changes in the position of bugs and the order of processes. Further, in our code-to-code translation, the mechanism for making and certifying promises and reservations is isolated in one module, and can easily be changed to cover different variants of the promising semantics.

For lack of space, detailed proofs can be found in [5].

## 2 Preliminaries

In this section, we introduce the notation that will be used throughout.

**Notations.** Given two natural numbers $i, j \in \mathbb{N}$ s.t. $i \leq j$, we use $[i, j]$ to denote $\{k \mid i \leq k \leq j\}$. Let $A$ and $B$ be two sets. We use $f : A \rightarrow B$ to denote that $f$ is a function from $A$ to $B$. We define $f[a \mapsto b]$ to be the function $f'$ s.t. $f'(a) = b$ and $f'(a') = f(a')$ for all $a' \neq a$. For a binary relation $R$, we use $[R]^*$ to denote its reflexive and transitive closure. Given an alphabet $\Sigma$, we use $\Sigma^*$ (resp. $\Sigma^+$) to denote the set of possibly empty (resp. non-empty) finite words (also called simple words) over $\Sigma$. A higher order word over $\Sigma$ is an element of $(\Sigma^*)^*$ (i.e., word of words). Let $w = a_1 a_2 \cdots a_n$ be a simple word over $\Sigma$, we use $|w|$ to denote the length of $w$. Given an index $i$ in $[1, |w|]$, we use $w[i]$ to denote the $i^{\text{th}}$ letter of $w$. Given two indices $i$ and $j$ s.t. $1 \leq i \leq j \leq |w|$, we use $w[i, j]$ to denote the word $a_i a_{i+1} \cdots a_j$. Sometimes, we see a word as a function from $[1, |w|]$ to $\Sigma$.

$$
\begin{array}{l}
\mathit{Prog} ::= \texttt{var}\ x^* (\texttt{proc}\ p || \ldots || \texttt{proc}\ p) \\
\texttt{proc}\ p ::= \mathsf{Reg}(p)\ \mathfrak{i}^* \\
\mathfrak{i} ::= \lambda : \mathfrak{s} \\
\mathfrak{s} \in \mathsf{St} ::= \\
\quad \mathsf{skip} \quad | s; s \quad | \texttt{assume}(x = e) \\
\quad | \texttt{do}\ s^*\ \texttt{while}\ e \quad | \texttt{while}\ e\ \texttt{do}\ s^* \texttt{done} \\
\quad | \texttt{if}\ e\ \texttt{then}\ s\ \texttt{else}\ s \\
\quad | \$r := e \quad | \$r := x^o \quad | x^o := \$r \\
\quad | \$r := \mathbf{FADD}^{o,o}(x, v) \\
\quad | \$r := \mathbf{CAS}^{o,o}(x, v, v) \quad | \mathsf{SC\text{-}fence} \\
o \in \mathsf{Mode} ::= \mathsf{rlx} | \mathsf{ra}
\end{array}
$$

Fig. 1: Syntax of programs.

**Program Syntax.** The simple programming language we use is described in Figure 1. A program *Prog* consists of a set $\mathsf{Loc}$ of (global) variables or memory locations, and a set $\mathcal{P}$ of processes. Each process $p$ declares a set $\mathsf{Reg}\,(p)$ of (local) *registers* followed by a sequence of labeled instructions. We assume that these sets of registers are disjoint and we use $\mathsf{Reg} := \cup_p \mathsf{Reg}\,(p)$ to denote their union. We assume also a (potentially unbounded) data domain $\mathsf{Val}$ from which the registers and locations take values. All locations and registers are assumed to be initialized with the special value $0 \in \mathsf{Val}$ (if not mentioned otherwise). An instruction $\mathfrak{i}$ is of the form $\lambda : \mathfrak{s}$ where $\lambda$ is a unique label and $\mathfrak{s}$ is a statement. We use $\mathbb{L}_p$ to denote the set of all labels of the process $p$, and $\mathbb{L} = \bigcup_{p \in \mathcal{P}} \mathbb{L}_p$ the set of all labels of all processes. We assume that the execution of the process $p$ starts always with a unique initial instruction labeled by $\lambda^p_{\text{init}}$.

A write instruction is of the form $x^o = \$r$ assigns the value of register $\$r$ to the location $x$, and $o$ denotes the access mode. If $o = \mathsf{rlx}$, the write is a *relaxed* write, while if $o = \mathsf{ra}$, it is a *release* write. A read instruction $\$r = x^o$ reads the value of the location $x$ into the local register $\$r$. Again, if the access mode $o = \mathsf{rlx}$, it is a *relaxed* read, and if $o = \mathsf{ra}$, it is an *acquire* read. Atomic updates or RMW instructions are either compare-and-swap ($\mathbf{CAS}^{o_r,o_w}$) or $\mathbf{FADD}^{o_r,o_w}$. Both have a pair of accesses ($o_r, o_w \in \{\mathsf{rel}, \mathsf{acq}, \mathsf{rlx}\}$) to the same location – a read followed by a write. Following [22], $\mathbf{FADD}(x, v)$ stores the value of $x$ into a register $\$r$, and adds $v$ to $x$, while $\mathbf{CAS}(x, v_1, v_2)$ compares an expected value $v_1$ to the value in $x$, and if the values are same, sets the value of $x$ to $v_2$. The old value of $x$ is then stored in $\$r$. A *local* assignment instruction $\$r = e$ assigns to the register $\$r$ the value of $e$, where $e$ is an expression over a set of operators, constants as well as the contents of the registers of the current process, but not referring to the set of locations. The fence instruction SC-fence is used to enforce sequential consistency if it is placed between two memory access operations. For simplicity, we will write $\texttt{assume}(x = e)$ instead of $\$r = x; \texttt{assume}(\$r = e)$. This notation is extended in the straightforward manner to conditional statements.

## 3 The Promising Semantics

In this section, we recall the promising semantics [22]. We present here PS 2.0 with three memory accesses, *relaxed*, *release writes* (`rel`) and *acquire reads* (`acq`).

Read-modify-writes (RMW) instructions have two access modes - one for read and one for write. We keep aside the release and acquire fences (and subsequent access modes), since they do not affect the results of this paper.

**Timestamps.** PS 2.0 uses timestamps to maintain a total order over all the writes to the same variable. We assume an infinite set of timestamps $\mathsf{Time}$, densely totally ordered by $\leq$, with 0 being the minimum element. A *view* is a timestamp function $V : \mathsf{Loc} \to \mathsf{Time}$ that records the largest known timestamp for each location. Let $\mathbb{T}$ be the set containing all the timestamp functions, along with the special symbol $\perp$. Let $V_{\text{init}}$ represent the initial view where all locations are mapped to 0. Given two views $V$ and $V'$, we use $V \leq V'$ to denote that $V(x) \leq V'(x)$ for $x \in \mathsf{Loc}$. The merge operation $\sqcup$ between two views $V$ and $V'$ returns the pointwise maximum of $V$ and $V'$, i.e., $(V \sqcup V')(y)$ is the maximum of $V(y)$ and $V'(y)$. Let $\mathcal{I}$ denote the set of all intervals over $\mathsf{Time}$. The timestamp intervals in $\mathcal{I}$ have the form $(f, t]$ where either $f = t = 0$ or $f < t$, with $f, t \in \mathsf{Time}$. Given an interval $I = (f, t] \in \mathcal{I}$, $I.\mathtt{frm}$ and $I.\mathtt{to}$ denote $f, t$ respectively.

**Memory.** In PS 2.0, the memory is modelled as a set of concrete *messages* (which we just call messages), and *reservations*. Each message represents the effect of a write or a RMW operation and each reservation is a timestamp interval reserved for future use. In more detail, a message $m$ is a tuple $(x, v, (f, t], V)$ where $x \in \mathsf{Loc}$, $v \in \mathsf{Val}$, $(f, t] \in \mathcal{I}$ and $V \in \mathbb{T}$. A reservation $r$ is a tuple $(x, (f, t])$. Note that a reservation, unlike a message, does not commit to any particular value. We use $m.\mathsf{loc}$ ($r.\mathsf{loc}$), $m.\mathtt{val}$, $m.\mathtt{to}$ ($r.\mathtt{to}$), $m.\mathtt{frm}$ ($r.\mathtt{frm}$) and $m.\mathsf{View}$ to denote respectively $x$, $v$, $t$, $f$ and $V$. Two elements (either messages or reservations) are said to be *disjoint* ($m_1 \# m_2$) if they concern different variables ($m_1.\mathsf{loc} \neq m_2.\mathsf{loc}$) or their intervals do not overlap ($m_1.\mathtt{to} \leq m_2.\mathtt{frm} \vee m_1.\mathtt{frm} \geq m_2.\mathtt{to}$). Two sets of elements $M, M'$ are disjoint, denoted $M \# M'$, if $m \# m'$ for every $m \in M, m' \in M'$. Two elements $m_1, m_2$ are *adjacent* denoted $\mathsf{Adj}(m_1, m_2)$ if $m_1.\mathsf{loc} = m_2.\mathsf{loc}$ and $m_1.\mathtt{to} = m_2.\mathtt{frm}$. A memory $M$ is a set of pairwise disjoint messages and reservations. Let $\widetilde{M}$ be the subset of $M$ containing only messages (no reservations). For a location $x$, let $M(x)$ be $\{m \in M \mid m.\mathsf{loc} = x\}$. Given a view $V$ and a memory $M$, we say $V \in M$ if $V(x) = m.\mathtt{to}$ for some message $m \in \widetilde{M}$ for every $x \in \mathsf{Loc}$. Let $\mathbb{M}$ denote the set of all memories.

**Insertion into Memory.** Following [22], a memory $M$ can be extended with a *message* (due to the execution of a write/RMW instruction) or a *reservation* $m$ with $m.\mathsf{loc} = x$, $m.\mathtt{frm} = f$ and $m.\mathtt{to} = t$ in a number of ways:

*Additive insertion* $M \overset{A}{\hookleftarrow} m$ is defined only if (1) $M \# \{m\}$; (2) if $m$ is a message, then no message $m' \in M$ has $m'.\mathsf{loc} = x$ and $m'.\mathtt{frm} = t$; and (3) if $m$ is a reservation, then there exists a message $m' \in \widetilde{M}$ with $m'.\mathsf{loc} = x$ and $m'.\mathtt{to} = f$. The extended memory $M \overset{A}{\hookleftarrow} m$ is then $M \cup \{m\}$.

*Splitting insertion* $M \overset{S}{\hookleftarrow} m$ is defined if $m$ is a message, and, if there exists a message $m' = (x, v', (f, t'], V)$ with $t < t'$ in $M$. Then $M$ is updated to $M \overset{S}{\hookleftarrow} m = (M \backslash \{m'\} \cup \{m, (x, v', (t, t'], V)\})$.

*Lowering Insertion* $M \overset{L}{\hookleftarrow} m$ is only defined if there exists $m'$ in $M$ that is identical to $m = (x, v, (f, t], V)$ except for $m.\mathsf{View} \leq m'.\mathsf{View}$. Then, $M$ is updated to $M \overset{L}{\hookleftarrow} m = M \backslash \{m'\} \cup \{m\}$.

**Transition System of a Process.** Given a process $p \in \mathcal{P}$, a state $\sigma$ of $p$ is defined by a pair $(\lambda, R)$ where $\lambda \in \mathbb{L}$ is the label of the next instruction to be executed by $p$ and $R : \mathsf{Reg} \to \mathsf{Val}$ maps each register of $p$ to its current value. (Observe that we use the set of all labels $\mathbb{L}$ (resp. registers $\mathsf{Reg}$) instead of $\mathbb{L}_p$ (resp. $\mathsf{Reg}\,(p)$) in the definition of $\sigma$ just for the sake of simplicity.) Transitions between the states of $p$ are of the form $(\lambda, R) \underset{p}{\overset{t}{\Rightarrow}} (\lambda', R')$ with $t$ is on one of the following forms: $\epsilon$, $\mathtt{rd}(o, x, v)$, $\mathtt{wt}(o, x, v)$, $\mathtt{U}(o_r, o_w, x, v_r, v_w)$, and $\mathsf{SC\text{-}fence}$. A transition of the form $(\lambda, R) \xRightarrow[p]{\mathtt{rd}(o,x,v)} (\lambda', R')$ denotes the execution of a read instruction of the form $\$r = x^o$ labeled by $\lambda$ where (1) $\lambda'$ is the label of the next instructions that can be executed after the instruction labelled by $\lambda$, and (2) $R'$ is the mapping that results from updating the value of the register $\$r$ in $R$ to $v$. The transition relation $(\lambda, R) \underset{p}{\overset{t}{\Rightarrow}} (\lambda', R')$ is defined in similar manner for the other cases of $t$ where $\mathtt{wt}(o, x, v)$ stands for a write instruction that writes the value $v$ to $x$, $\mathtt{U}(o_r, o_w, x, v_r, v_w)$ stands for a RMW that reads the value $v_r$ from $x$ and write $v_w$ to it, $\mathsf{SC\text{-}fence}$ stands for a $\mathsf{SC\text{-}fence}$ instruction, and $\epsilon$ stands for the execution of the other local instructions. Observe that $o, o_r, o_w$ are the access modes which can be $\mathtt{rlx}$ or $\mathtt{ra}$. We use $\mathtt{ra}$ for both release and acquire. Finally, we use $(\lambda, R) \underset{p}{\overset{t}{\rightarrow}} (\lambda', R')$, with $t \neq \epsilon$, to denote that $(\lambda, R) \underset{p}{\overset{\epsilon}{\Rightarrow}} \sigma_1 \underset{p}{\overset{\epsilon}{\Rightarrow}} \cdots \underset{p}{\overset{\epsilon}{\Rightarrow}} \sigma_n \underset{p}{\overset{t}{\Rightarrow}} \sigma_{n+1} \underset{p}{\overset{\epsilon}{\Rightarrow}} \cdots \underset{p}{\overset{\epsilon}{\Rightarrow}} (\lambda', R')$.

**Machine States.** A machine state $\mathcal{MS}$ is a tuple $((\mathsf{J}, \mathsf{R}), \mathsf{VS}, \mathsf{PS}, M, G)$, where $\mathsf{J} : \mathcal{P} \mapsto \mathbb{L}$ maps each process $p$ to the label of the next instruction to be executed, $\mathsf{R} : \mathsf{Reg} \to \mathsf{Val}$ maps each register to its current value, $\mathsf{VS} = \mathcal{P} \to \mathbb{T}$ is the process view map, which maps each process to a view, $M$ is a memory and $PS : \mathcal{P} \mapsto \mathbb{M}$ maps each process to a set of messages (called *promise* set), and $G \in \mathbb{T}$ is the global view (that will be used by SC fences). We use $\mathcal{C}$ to denote the set of all machine states. Given a machine state $\mathcal{MS} = ((\mathsf{J}, \mathsf{R}), \mathsf{VS}, \mathsf{PS}, M, G)$ and a process $p$, let $\mathcal{MS}{\downarrow}p$ denote $(\sigma, \mathsf{VS}(p), \mathsf{PS}(p), M, G)$, with $\sigma = (\mathsf{J}(p), \mathsf{R}(p))$, (i.e., the projection of the machine state to the process $p$). We call $\mathcal{MS}{\downarrow}p$ the process configuration. We use $\mathcal{C}_p$ to denote the set of all process configurations.

The initial machine state $\mathcal{MS}_{\text{init}} = ((\mathsf{J}_{\text{init}}, \mathsf{R}_{\text{init}}), \mathsf{VS}_{\text{init}}, \mathsf{PS}_{\text{init}}, M_{\text{init}}, G_{\text{init}})$ is one where: (1) $\mathsf{J}_{\text{init}}(p)$ is the label of the initial instruction of $p$; (2) $\mathsf{R}_{\text{init}}(\$r) = 0$ for every $\$r \in \mathsf{Reg}$; (3) for each $p$, $\mathsf{VS}(p) = V_{\text{init}}$ as the initial view (that maps each location to the timestamp 0); (4) for each $p$, the set of promises $\mathsf{PS}_{\text{init}}(p)$ is empty; (5) the initial memory $M_{\text{init}}$ contains exactly one initial message $(x, 0, (0, 0], V_{\text{init}})$ per location $x$; and (6) the initial global view maps each location to 0.

**Transition Relation.** We first describe the transition $(\sigma, V, P, M, G) \underset{p}{\rightarrow} (\sigma', V', P', M', G')$ between process configurations in $\mathcal{C}_p$ from which we induce the transition relation between machine states.

**Memory Helpers**

$$\frac{\text{(MEMORY : NEW)}}{(P, M) \xrightarrow{m} \left(P', M \overset{A}{\hookleftarrow} m\right)}$$

$$\frac{\text{MEMORY FULFIL} \quad \hookleftarrow \in \left\{\overset{S}{\hookleftarrow}, \overset{L}{\hookleftarrow}\right\}, P' = P \hookleftarrow m, M' = M \hookleftarrow m}{(P, M) \xrightarrow{m} (P' \backslash \{m\}, M')}$$

**Process Helpers**

$$\frac{m = (x, -, (-, t], K) \in M \quad V(x) \leq t \quad o = \texttt{rlx} \Rightarrow V' = V[x \mapsto t] \quad o = \texttt{ra} \Rightarrow V' = V[x \mapsto t] \sqcup K}{V \xrightarrow[\text{rd}]{o,m} V'}$$

$$\frac{m = (x, -, (-, t], K) \in M, V(x) < t \quad o = \texttt{rlx} \Rightarrow K = \bot, o = \texttt{ra} \Rightarrow P(x) = \emptyset \wedge K = V' \quad (P, M) \xrightarrow{m} (P', M') \quad V' = V[x \mapsto t]}{(V, P, M) \xrightarrow[\text{wt}]{o,m} (V', P', M')}$$

**Process Steps**

Read

$$\frac{\sigma \xrightarrow[p]{rd(o,x,v)} \sigma' \quad m = (x, v, (-, -],\ -), \quad V \xrightarrow[\text{rd}]{o,m} V'}{(\sigma, V, P, M, G) \underset{p}{\rightarrow} (\sigma', V', P, M, G)}$$

Write

$$\frac{\sigma \xrightarrow[p]{wt(o,x,v)} \sigma' \quad m = (x, v, (-, -], -), (V, P, M) \xrightarrow[\text{wt}]{o,m} (V', P', M')}{(\sigma, V, P, M, G) \underset{p}{\rightarrow} (\sigma', V', P', M', G)}$$

SC-fence

$$\frac{\sigma \xrightarrow[p]{\text{SC-fence}} \sigma'}{(\sigma, V, P, M, G) \underset{p}{\rightarrow} (\sigma', V \sqcup G, P, M, G \sqcup V)}$$

Promise

$$\frac{m = (-, -, (-, -], K), \quad M' = M \overset{A}{\hookleftarrow} m,\ K \in M'}{(\sigma, V, P, M, G) \underset{p}{\rightarrow} \left(\sigma, V, P \overset{A}{\hookleftarrow} m, M', G\right)}$$

Update

$$\frac{\sigma \xrightarrow[p]{U(o_r, o_w, x, v_r, v_w)} \sigma'', m_r = (x, v_r,\ (-, t],\ -),\ m_w = (x, v_w, (t, -], -), \quad V \xrightarrow[\text{rd}]{o_r, m_r} V'',\ (V'', P, M) \xrightarrow[\text{wt}]{o_w, m_w} (V', P', M')}{(\sigma, V, P, M, G) \underset{p}{\rightarrow} (\sigma', V', P', M', G)}$$

Fig. 2: A subset of PS 2.0 inference rules at the process level.

*Process Relation.* The formal definition of $\underset{p}{\rightarrow}$ is given in Figure 2. Below, we explain these inference rules. Note that the full set of rules can be found in [5].
*Read* A process $p$ can read from $M$ by observing a message $m = (x, v, (f, t], K)$ if $V(x) \leq t$ (i.e., $p$ must not be aware of a later message for $x$). In case of a relaxed read $\texttt{rd}(\texttt{rlx}, x, v)$, the process view of $x$ is updated to $t$, while for an acquire read $\texttt{rd}(\texttt{ra}, x, v)$, the process view is updated to $V[x \mapsto t] \sqcup K$. The global memory $M$, the set of promises $P$, and the global view $G$ remain the same.

*Write.* A process can add a fresh message to the memory (MEMORY : NEW) or fulfil an outstanding promise (MEMORY : FULFILL). The execution of a write ($\texttt{wt}(\texttt{rlx}, x, v)$) results in a message $m$ with location $x$ along with a timestamp interval $(-, t]$. Then, the process view for $x$ is updated to $t$. In case of a release write ($\texttt{wt}(\texttt{ra}, x, v)$) the updated process view is also attached to $m$, and ensures that the process does not have an outstanding promise on $x$. (MEMORY : FULFILL) allows to split a promise interval or lower its view before fulfilment.

*Update.* When a process performs a RMW, it first reads a message $m = (x, v, (f, t], K)$ and then writes an update message with $\texttt{frm}$ timestamp equal to $t$; that is, a message of the form $m' = (x, v', (t, t'], K')$. This forbids any other write to be placed between $m$ and $m'$. The access modes of the reads and writes in the update follow what has been described for the read and write above.

*Promise, Reservation and Cancellation.* A process can non-deterministically *promise* future writes which are not release writes. This is done by adding a message $m$ to the memory $M$ s.t. $m\#M$ and to the set of promises $P$. Later, a relaxed write instruction can fulfil an existing promise. Recall that the execution of a release write requires that the set of promises to be empty and thus it can not be used to fulfil a promise. In the reserve step, the process reserves a timestamp interval to be used for a later RMW instruction reading from a certain message without fixing the value it will write. A reservation is added both to the memory and the promise set. The process can drop the reservation from both sets using the cancel step in non-deterministic manner.

*SC fences.* The process view $V$ is merged with the global view $G$, resulting in $V \sqcup G$ as the updated process view and global view.

**Machine Relation.** We are ready now to define the induced transition relation between machine states. For machine states $\mathcal{MS} = ((J, R), VS, PS, M, G)$ and $\mathcal{MS}' = ((J', R'), VS', PS', M', G')$, we write $\mathcal{MS} \underset{p}{\rightarrow} \mathcal{MS}'$ iff (1) $\mathcal{MS}{\downarrow}p \underset{p}{\rightarrow} \mathcal{MS}{\downarrow}p$ and $(J(p'), VS(p'), PS(p')) = (J'(p'), VS'(p'), PS'(p'))$ for all $p' \neq p$.

**Consistency.** According to Lee et al. [22], there is one final requirement on machine states called *consistency*, which roughly states that, from every encountered machine state, all the messages promised by a process $p$ can be *certified* (i.e., made fulfillable) by executing $p$ on its own from a certain future memory (called capped memory), i.e., extension of the memory with additional reservation. Before defining consistency, we need to introduce capped memory.

*Cap View, Cap Message and Capped Memory.* The last element of a memory $M$ with respect to a location $x$, denoted by $\overline{m}_{M,x}$, is an element from $M(x)$ with the highest timestamp among all elements of $M(x)$ and is defined as $\overline{m}_{M,x} = \max_{m \in M(x)} m.\mathtt{to}$. The *cap view* of a memory $M$, denoted by $\widehat{V}_M$, is the view which assigns to each location $x$, the $\mathtt{to}$ timestamp in the message $\overline{m}_{\widetilde{M},x}$. That is, $\widehat{V}_M = \lambda x.\overline{m}_{\widetilde{M},x}.\mathtt{to}$. Recall that $\widetilde{M}$ denote the subset of $M$ containing only messages (no reservations). The *cap message* of a memory $M$ with respect to a location $x$, is given by $\widehat{m}_{M,x} = (x, \overline{m}_{\widetilde{M},x}.\mathtt{val}, (\overline{m}_{M,x}.\mathtt{to}, \overline{m}_{M,x}.\mathtt{to} + 1], \widehat{V}_M)$.

Then, the capped memory of a memory $M$, wrt. a set of promises $P$, denoted by $\widehat{M}_P$, is an extension of $M$, defined as: (1) for every $m_1, m_2 \in M$ with $m_1.\mathsf{loc} = m_2.\mathsf{loc}$, $m_1.\mathtt{to} < m_2.\mathtt{frm}$, and there is no message $m' \in M(m_1.\mathsf{loc})$ such that $m_1.\mathtt{to} < m'.\mathtt{to} < m_2.\mathtt{to}$, we include a reservation $(m_1.\mathsf{loc}, (m_1.\mathtt{to}, m_2.\mathtt{frm}])$ in $\widehat{M}_P$, and (2) we include a cap message $\widehat{m}_{M,x}$ in $\widehat{M}_P$ for every variable $x$ unless $\overline{m}_{M,x}$ is a reservation in $P$.

*Consistency.* A machine state $\mathcal{MS} = ((J, R), VS, PS, M, G)$ is *consistent* if every process $p$ can certify/fulfil all its promises from the capped memory $\widehat{M}_{PS(p)}$, i.e., $((J, R), VS, PS, \widehat{M}_{PS(p)}, G)\ [\underset{p}{\rightarrow}]^*\ ((J', R'), VS', PS', M', G')$ with $PS'(p) = \emptyset$.

**The Reachability Problem in PS 2.0.** A run of *Prog* is a sequence of the form: $\mathcal{MS}_0 [\xrightarrow[p_{i_1}]{}]^* \mathcal{MS}_1 [\xrightarrow[p_{i_2}]{}]^* \mathcal{MS}_2 [\xrightarrow[p_{i_3}]{}]^* \ldots [\xrightarrow[p_{i_n}]{}]^* \mathcal{MS}_n$ where $\mathcal{MS}_0 = \mathcal{MS}_{\text{init}}$ is the initial machine state and $\mathcal{MS}_1, \ldots, \mathcal{MS}_n$ are consistent machine states. Then, $\mathcal{MS}_0, \ldots, \mathcal{MS}_n$ are said to be reachable from $\mathcal{MS}_{\text{init}}$.

Given an instruction label function $J : \mathcal{P} \to \mathbb{L}$ that maps each process $p \in \mathcal{P}$ to an instruction label in $\mathbb{L}_p$, the *reachability* problem asks whether there exists a machine state of the form $((J, R), V, P, M, G)$ that is reachable from $\mathcal{MS}_{\text{init}}$. A positive answer to this problem means that $J$ is reachable in *Prog* in PS 2.0.

## 4 Undecidability of Consistent Reachability in PS 2.0

The reachability problem is undecidable for PS 2.0 even for finite-state programs. The proof is by a reduction from Post's Correspondence Problem (PCP) [28]. A PCP instance consists of two sequences $u_1, \ldots, u_n$ and $v_1, \ldots, v_n$ of non-empty words over some alphabet $\Sigma$. Checking whether there exists a sequence of indices $j_1, \ldots, j_k \in \{1, \ldots, n\}$ s.t. $u_{j_1} \ldots u_{j_k} = v_{j_1} \ldots v_{j_k}$ is undecidable. Our proof works with the fragment of PS 2.0 having only relaxed (`rlx`) memory accesses and crucially uses unboundedly many promises to ensure that a process cannot skip any writes made by another process. We construct a concurrent program with two processes $p_1$ and $p_2$ over a finite data domain. The code of $p_1$ is split into two modes: a generation mode and a validation mode by a `if` and its `else` branch. The `if` branch is entered when the value of a boolean location *validate* is 0 (its initial value). We show that reaching the instructions annotated by // and // in $p_1, p_2$ is possible iff the PCP instance has a solution. We give below an overview of the execution steps leading to the annotated instructions.

- Process $p_1$ promises to write letters of $u_i$ (one by one) to a location $x$, and the respective indices $i$ to a location *index*. The number of made promises is arbitrary, since it depends on the length of the PCP solution. Observe that the sequence of promises made to the variable *index* corresponds to the guessed solution of the PCP problem.
- Before switching out of context, $p_1$ certifies its promise using the `if` branch which consists of a loop that non-deterministically chooses an index $i$ and writes $i$ to *index* and $u_i$ to $x$. The promises of $p_1$ are as yet not fulfilled; this happens in the `else` branch of $p_1$, when it writes the promised values.
- $p_2$ reads from the sequences of promises written to $x$ and *index* and copies them (one by one) to variables $y$ and $index'$ respectively. Then, $p_2$ sets *validate* to 1 and reaches //.
- The `else` branch in $p_1$ is enabled at this point, where $p_1$ reads the sequence of indices from $index'$, and each time it reads an index $i$ from $index'$, it checks that it can read the sequence of letters of $v_i$ from $y$.
- $p_1$ copies the sequence of observed values from $y$ and $index'$ back to $x$ and *index* respectively. To fulfil the promises, it is crucial that the sequence of read values from $index'$ (resp. $y$) is the same as the sequence of promised values to *index* (resp. $x$). Since $y$ holds a sequence $v_{i_1} \ldots v_{i_k}$, the promises are fulfilled if and only if this sequence is same as the promised sequence $u_{i_1} \dots u_{i_k}$. This happens only when $i_1, \dots, i_k$ is a PCP solution.
– At the end of promise fulfilment, $p_1$ reaches //.

Our undecidability result is also *tight* in the sense that the reachability problem becomes decidable when we restrict ourselves to machine states where the number of promises is bounded. Further, our proof is robust: it goes through for PS 1.0 [16]. Let us call the fragment of PS 2.0 with only `rlx` memory accesses PS 2.0-rlx.

**Theorem 1.** *The reachability problem for concurrent programs over a finite data domain is undecidable under PS 2.0-rlx.*

## 5 Decidable Fragments of PS 2.0

Since keeping `ra` memory accesses renders the reachability problem undecidable [1] and so does having unboundedly many promises when having `rlx` memory accesses (Theorem 1), we address in this section the decidability problem for PS 2.0-rlx with a bounded number of promises in any reachable configuration. Bounding the number of promises in any reachable machine state does not imply that the total number of promises made during that run is bounded. Let bdPS 2.0-rlx represent the restriction of PS 2.0-rlx to boundedly many promises where the number of promises in each reachable machine state is smaller or equal to a given constant. Notice that the fragment bdPS 2.0-rlx subsumes the relaxed fragment of the RC11 model [20,16].We assume here a finite data domain.

To establish the decidability of the reachability of bdPS 2.0-rlx, we introduce an alternate memory model for concurrent programs called LoHoW (for "lossy higher order words"). We present the operational semantics of LoHoW, and show that (1) PS 2.0-rlx is reachability equivalent to LoHoW, (2) under the bounded promise assumption, reachability is decidable in LoHoW (hence, bdPS 2.0-rlx).

**Introduction to LoHoW.** Given a concurrent program *Prog*, a *state* of LoHoW maintains a collection of higher order words, one per location of *Prog*, along with the states of all processes. The higher order word $\mathsf{HW}_x$ corresponding to the location $x$ is a word of simple words, representing the sub memory $M(x)$ in PS 2.0-rlx. Each simple word in $\mathsf{HW}_x$ is an ordered sequence of "memory types", that is, messages or promises in $M(x)$, maintained in the order of their `to` timestamps in the memory. The word order between memory types in $\mathsf{HW}_x$ represents the order induced by time stamps between memory types in $M(x)$. The key information to encode in each memory type of $\mathsf{HW}_x$ is: (1) is it a message (msg) or a promise (`prm`) in $M(x)$, (2) the process ($p$) which added it to $M(x)$, the value (`val`) it holds, (3) the set $S$ (called pointer set) of processes that have seen this memory type in $M(x)$ and (4) whether the adjacent time interval to the right of this memory type in $M(x)$ has been reserved by some process.

**Memory Types.** To keep track of (1-4) above, a *memory type* is an element of $\Sigma \cup \Gamma$ with, $\Sigma = \{\mathsf{msg}, \mathtt{prm}\} \times \mathsf{Val} \times \mathcal{P} \times 2^{\mathcal{P}}$ (for 1-3) and $\Gamma = \{\mathsf{msg}, \mathtt{prm}\} \times \mathsf{Val} \times \mathcal{P} \times 2^{\mathcal{P}} \times \mathcal{P}$ (for 4). We write a memory type as $(r, v, p, S, \underline{?})$. Here $r$ represents either msg (message) or prm (promise) in $M(x)$, $v$ is the value, $p$ is the process that added the message/promise, $S$ is a *pointer set* of processes whose local view (on $x$) agrees with the to timestamp of the message/promise. If the type $\in \Gamma$, the fifth component ($\underline{?}$) is the process id that has reserved the time slot right-adjacent to the message/promise. $\underline{?}$ is a wildcard that may (or not) be matched.

**Simple Words.** A simple word $\in \Sigma^*\#(\Sigma \cup \Gamma)$, and each $\mathsf{HW}_x$ is a word $\in (\Sigma^*\#(\Sigma \cup \Gamma))^+$. # is a special symbol not in $\Sigma \cup \Gamma$, which separates the last symbol from the rest of the simple word. Consecutive symbols of $\Sigma$ in a simple word in $\mathsf{HW}_x$ represent adjacent messages/promises in $M(x)$ and are hence unavailable for a RMW. # does not correspond to any element from the memory, and is used to demarcate the last symbol of the simple word.

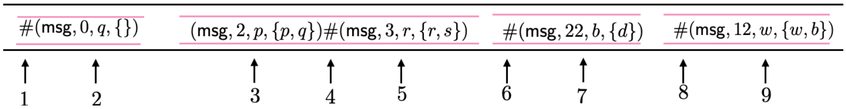

Fig. 3: A higher order word $\mathsf{HW}$ (black) with four embedded simple words (pink).

**Higher order words**. A *higher order word* is a sequence of simple words. Figure 3 depicts a higher order word with four simple words. We use a left to right order in both simple words and higher order words. Furthermore, we extend in the straightforward manner the classical word indexation strategy to higher order words. For example, the symbol at the third position of the higher order word $\mathsf{HW}$ in Figure 3 is $\mathsf{HW}[3] = (\mathsf{msg}, 2, p, \{p, q\})$. A higher order word $\mathsf{HW}$ is *well-formed* iff for every $p \in \mathcal{P}$, there is a unique position $i$ in $\mathsf{HW}$ having $p$ in its pointer set; that is, $\mathsf{HW}[i]$ is of the form $(-, -, -, S, \underline{?}) \in \Sigma \cup \Gamma$ s.t. $p \in S$. The higher order word given in Figure 3 is well-formed. We will use $\mathsf{ptr}(p, \mathsf{HW})$ to denote the unique position $i$ in $\mathsf{HW}$ having $p$ in its pointer set. We assume that all the manipulated higher order words are well-formed.

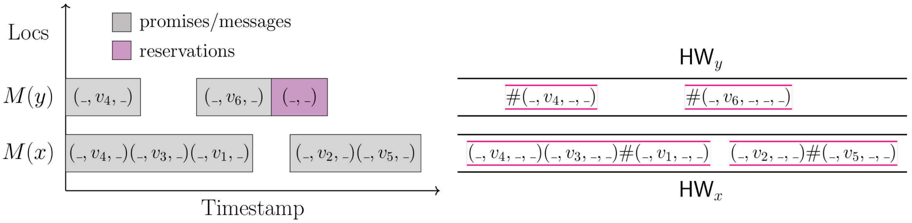

Fig. 4: Map from memories $M(x), M(y)$ to higher order words $\mathsf{HW}_x, \mathsf{HW}_y$.

Each higher order word $\mathsf{HW}_x$ represents the entire space $[0, \infty)$ of available timestamps in $M(x)$. Each simple word in $\mathsf{HW}_x$ represents a timestamp interval $(f, t]$, while consecutive simple words represent disjoint timestamp intervals (while preserving order). The memory types constituting each simple word take up *adjacent* timestamp intervals, spanning the timestamp interval of the simple word. The adjacency of timestamp intervals within simple words is used in RMW steps and reservations. The last symbol in a simple word denotes a message/promise which, (1) if in $\Sigma$, is available for a RMW, while (2) if in $\Gamma$, is unavailable for RMW since it is followed by a reservation. Symbols at positions other than the rightmost in a simple word, represent messages/promises which are not available for RMW. Figure 4 presents a mapping from a memory of PS 2.0-rlx to a collection of higher order words (one per location) in LoHoW.

**Initializing higher order words.** For each location $x \in \mathsf{Loc}$, the initial higher order word $\mathsf{HW}_x^{\text{init}}$ is defined as $\#(\mathsf{msg}, 0, p_1, \mathcal{P})$, where $\mathcal{P}$ is the set of all processes and $p_1$ is some process in $\mathcal{P}$. The set of all higher order words $\mathsf{HW}_x^{\text{init}}$ for all locations $x$ represents the initial memory of PS 2.0-rlx where all locations have value 0, and all processes are aware of the initial message.

**Simulating PS 2.0 Memory Operations in LoHoW.** In the following, we describe how to handle PS 2.0-rlx instructions in LoHoW. Since we only have the `rlx` mode, we denote Reads, Writes and RMWs as $\mathtt{wt}(x, v)$, $\mathtt{rd}(x, v)$ and $\mathtt{U}(x, v_r, v_w)$, dropping the modes.

Reads. To simulate a $\mathtt{rd}(x, v)$ by a process $p$ in LoHoW, we need an index $j \geq \mathsf{ptr}(p, \mathsf{HW}_x)$ in $\mathsf{HW}_x$ such that $\mathsf{HW}_x[j]$ is a memory type with value $v$ of the form $(-, v, -, S', \underline{?})$ ($\underline{?}$ denotes that the type is either from $\Sigma$ or $\Gamma$). The read is simulated by adding $p$ to the set $S'$ and removing it from its previous set.

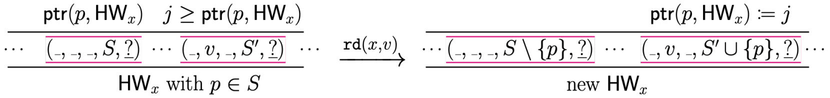

Fig. 5: Transformation of $\mathsf{HW}_x$ on a read. ($\underline{?}$ denotes that type is from $\Sigma$ or $\Gamma$)

Writes. A $\mathtt{wt}(x, v)$ by a process $p$ (writing $v$ to $x$) is simulated by adding a new $\mathsf{msg}$ type in $\mathsf{HW}_x$ with a timestamp higher than the view of $p$ for $x$: (1) add the simple word $(\mathsf{msg}, v, p, \{p\})$ to the right of $\mathsf{ptr}(p, \mathsf{HW}_x)$ or (2) there is $\alpha \in \Sigma$ such that the word $w\#\alpha$ is in $\mathsf{HW}_x$ to the right of $\mathsf{ptr}(p, \mathsf{HW}_x)$. Modify $w\#\alpha$ to get $w\alpha\#(\mathsf{msg}, v, p, \{p\})\cdot$. Remove $p$ from its previous pointer set.

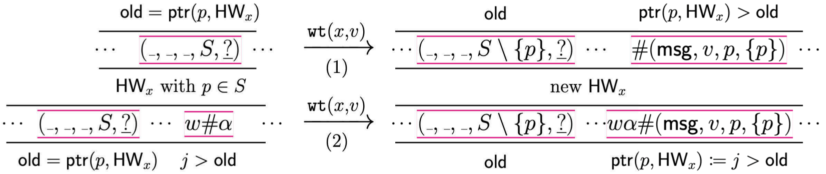

Fig. 6: Transformation of $\mathsf{HW}_x$ on a write. ($\underline{?}$ denotes that type is from $\Sigma$ or $\Gamma$).

RMWs. Capturing RMWs is similar to the execution of a read followed by a write. In PS 2.0-rlx, a process $p$ performing an RMW, reads from a message with a timestamp interval $(, t]$ and adds a message to $M(x)$ with timestamp interval $(t, -]$. Capturing RMWs needs higher order words. Consider a $\mathtt{U}(x, v_r, v_w)$ step by process $p$. Then, there is a simple word $\cdots\#(-, v_r, -, S)$ in $\mathsf{HW}_x$ having $(-, v_r, -, S)$ as the last memory type whose position is to the right of $\mathsf{ptr}(p, \mathsf{HW}_x)$. As usual, $p$ is removed from its pointer set, $\#(-, v_r, -, S)$ is replaced with $(-, v_r, -, S\backslash\{p\})\#$ and $(-, v_w, p, \{p\})$ is appended, resulting in extending $\cdots\#(-, v_r, -, S)$ to $\cdots(-, v_r, -, S\backslash\{p\})\#(-, v_w, p, \{p\})$.

Promises, Reservations and Cancellations. Handling promises made by a process $p$ in PS 2.0-rlx is similar to handling $\mathtt{wt}(x, v)$: we add the simple word $\#(\mathsf{prm}, v, p, \{\})$ in $\mathsf{HW}_x$ to the right of the position $\mathsf{ptr}(p, \mathsf{HW}_x)$, or append $(\mathtt{prm}, v, p, \{\})$ at the end of a simple word with a position larger than $\mathsf{ptr}(p, \mathsf{HW}_x)$. The memory type has tag $\mathtt{prm}$ (a promise), and the pointer set is empty (since making a promise does not lift the view of the promising process). Splitting the time interval of a promise is simulated in LoHoW by inserting a new memory type right before the corresponding promise memory type $(\mathtt{prm}, -, p, S)$, while fulfilment of a promise by a process $p$ results in replacing $(\mathtt{prm}, v, p, S)$ with $(\mathsf{msg}, v, p, S \cup \{p\})$.

In PS 2.0-rlx, a process $p$ makes a reservation by adding the pair $(x, (f, t])$ to the memory, given that there is a message/promise in the memory with timestamp interval $(-, f]$. In LoHoW this is captured by "tagging" the rightmost memory type (message/promise) in a simple word with the name of the process that makes the reservation. This requires us to consider the memory types from $\Gamma = \{\mathsf{msg}, \mathtt{prm}\} \times \mathsf{Val} \times \mathcal{P} \times 2^{\mathcal{P}} \times \mathcal{P}$ where the last component stores the process which made the reservation. Such a memory type always appears at the end of a simple word, and represents that the next timestamp interval adjacent to it has been reserved. Observe that nothing can be added to the right of a memory type of the form $(\mathsf{msg}, v, p, S, q)$. Thus, reservations are handled as follows.

(Res) Assume the rightmost symbol in a simple word as $(\mathsf{msg}, v, p, S)$. To capture the reservation by $q$, $(\mathsf{msg}, v, p, S)$ is replaced with $(\mathsf{msg}, v, p, S, q)$.

(Can) A cancellation is done by removing the last component $q$ from $(\mathsf{msg}, v, p, S, q)$ resulting in $(\mathsf{msg}, v, p, S)$.

**Certification** In PS 2.0-rlx, certification for a process $p$ happens from the capped memory, where intermediate time slots (other than reserved ones) are blocked, and any new message can be added only at the maximal timestamp. This is handled in LoHoW by one of the following: (1) Addition of new memory types is allowed only at the right end of any $\mathsf{HW}_x$, or (2) If the rightmost memory type in $\mathsf{HW}_x$ is of form $(-, v, -, -, q)$ with $q \neq p$ (a reservation by $q$), then the word $\#(\mathsf{msg}, v, q, \{\})$ is appended at end of $\mathsf{HW}_x$.

Memory is altered in PS 2.0-rlx during certification phase to check for promise fulfilment, and at the end of the certification phase, we resume from the memory which was there before. To capture this in LoHoW, we work on a duplicate of $(\mathsf{HW}_x)_{x \in \mathsf{Loc}}$ in the certification phase. Notice that the duplication allows losing non-deterministically, *empty memory types*: these are memory types whose pointer set is empty, as well as *redundant simple words*, which are simple words consisting entirely of empty memory types. This copy of $\mathsf{HW}_x$ is then modified during certification, and is discarded once we finish the certification phase.

### 5.1 Formal Model of LoHoW

In the following, we formally define LoHoW and state the equivalence of the reachability problem in PS 2.0-rlx and LoHoW. For a memory type $m = (r, v, p, S)$ (or $m = (r, v, p, S, q)$), we use $m.value$ to denote $v$. For a memory type $(r, v, p, S, \underline{?})$ and a process $p' \in \mathcal{P}$, we define the following: $\mathsf{add}(m, p') \equiv (r, v, p, S \cup \{p'\}, \underline{?})$ and $\mathsf{del}(m, p') \equiv (r, v, p, S \setminus \{p'\}, \underline{?})$. This corresponds to the addition/deletion of the process $p'$ to/from the set of pointers of $m$. Extending the above notation, given a higher order word $\mathsf{HW}$, a position $i \in \{1, \ldots, |\mathsf{HW}|\}$, and $p \in \mathcal{P}$ , we define the following: $\mathsf{add}(\mathsf{HW}, p, i) \equiv \mathsf{HW}[1, i-1] \cdot \mathsf{add}(\mathsf{HW}[i], p) \cdot \mathsf{HW}[i+1, |\mathsf{HW}|]$, $\mathsf{add}(\mathsf{HW}, p, i) \equiv \mathsf{HW}[1, i-1] \cdot \mathsf{add}(\mathsf{HW}[i], p) \cdot \mathsf{HW}[i+1, |\mathsf{HW}|]$, and $\mathsf{mov}(\mathsf{HW}, p, i) \equiv \mathsf{add}(\mathsf{del}(\mathsf{HW}, p), p, i)$. This corresponds to the addition/deletion/relocation of the pointer $p$ to/from the word $\mathsf{HW}[i]$.

**Insertion into higher order words.** A higher order word $\mathsf{HW}$ can be extended in position $1 \leq j \leq |\mathsf{HW}|$ with a memory type $m = (r, v, p, \{p\})$ as follows:

• *Insertion as a new simple word* is defined only if $\mathsf{HW}[j-1] = \#$ (i.e., the position $j$ is the end of a simple word). Let $\mathsf{HW}' = \mathsf{del}(\mathsf{HW}, p)$ (i.e., removing $p$ from its previous set of pointers). Then, the insertion of $m$ results in

$$\mathsf{HW} \overset{N}{\underset{j}{\hookleftarrow}} m \equiv \mathsf{HW}'[1, j] \cdot \underbrace{\#(r, v, p, \{p\})}_{\text{new simple word}} \cdot \mathsf{HW}'[j+1, |\mathsf{HW}|]$$

• *Insertion at the end of a simple word* is defined only if $\mathsf{HW}[j-1] = \#$ and $\mathsf{HW}[j] \in \Sigma$ (i.e., the last memory type in the simple word should be free from reservations). Let $\mathsf{HW}' = \mathsf{del}(\mathsf{HW}, p)$. For $\mathsf{HW}' = w_1 \cdot \# m' \cdot w_2$, and $|w_1 \cdot \# m'| = j$ the insertion of $m$ results in

$$\mathsf{HW} \overset{E}{\underset{j}{\hookleftarrow}} m \equiv w_1 \cdot m' \cdot \underbrace{\#(r, v, p, \{p\})}_{m \text{ extends } m'} \cdot w_2$$

• *Splitting a promise* is defined only if $m' = \mathsf{HW}[j]$ has form $(\mathtt{prm}, -, p, -, \underline{?})$ (i.e., the memory type at position $j$ is a promise). Let $\mathsf{HW}' = \mathsf{del}(\mathsf{HW}, p)$. Then,

$$\mathsf{HW} \overset{SP}{\underset{j}{\hookleftarrow}} m \equiv \begin{cases} \mathsf{HW}'[1, j-2] \cdot \underbrace{(r, v, p, \{p\}) \cdot \# m'}_{m \text{ splits } m'} \cdot \mathsf{HW}'[j+1, |\mathsf{HW}|] & \text{if } \mathsf{HW}'[j-1] = \# \\ \mathsf{HW}'[1, j-1] \cdot \underbrace{(r, v, p, \{p\}) \cdot m'}_{m \text{ splits } m'} \cdot \mathsf{HW}'[j+1, |\mathsf{HW}|] & \text{if } \mathsf{HW}'[j-1] \neq \# \end{cases}$$

Observe that in both cases we insert the new type $m$ just before position $j$.

• *Fulfilment of a promise* is defined only if $m' = \mathsf{HW}[j]$ is of the form $(\mathtt{prm}, v, p, S)$ or $(\mathtt{prm}, v, p, S, q)$. Let $\mathsf{HW}' = \mathsf{del}(\mathsf{HW}, p)$. Then, the extended higher order

$$\mathsf{HW} \overset{FP}{\underset{j}{\hookleftarrow}} m \equiv \mathsf{HW}'[1, j-1] \cdot \underbrace{(\mathsf{msg}, v, p, S \cup \{p\}, \underline{?})}_{m' \text{ is fulfilled by } p} \cdot \mathsf{HW}'[j+1, |\mathsf{HW}'|]$$

where $\underline{?}$ is $q$ if $m' = (\mathtt{prm}, v, p, S, q) \in \Gamma$ and is omitted if $m' = (\mathtt{prm}, v, p, S) \in \Sigma$.

**Making/Canceling a reservation.** A higher order word $\mathsf{HW}$ can also be modified by $p$ by making/cancelling a reservation at a position $1 \leq j \leq |\mathsf{HW}|$. We define the operation $\mathit{Make}(\mathsf{HW}, p, j)$ ($\mathit{Cancel}(\mathsf{HW}, p, j)$) that reserves (cancels) a time slot at $j$. $\mathit{Make}(\mathsf{HW}, p, j)$ (resp. $\mathit{Cancel}(\mathsf{HW}, p, j)$) is only defined if $\mathsf{HW}[j]$ is of the form $(r, v, q, S)$ (resp. $(r, v, q, S, p)$) and $\mathsf{HW}[j-1] = \#$. Then, we have $\mathit{Make}(\mathsf{HW}, p, j) \equiv \mathsf{HW}[1, j-1] \cdot (r, v, q, S, p) \cdot \mathsf{HW}[j+1, |\mathsf{HW}|]$ and $\mathit{Cancel}(\mathsf{HW}, p, j) \equiv \mathsf{HW}[1, j-1] \cdot (r, v, q, S) \cdot \mathsf{HW}[j+1, |\mathsf{HW}|]$.

**Process configuration in LoHoW.** A configuration of $p \in \mathcal{P}$ in LoHoW consists of a pair $(\sigma, \mathbf{HW})$ where (1) $\sigma$ is the process state maintaining the instruction label and the register values (see Subsection 3), and $\mathbf{HW}$ is a mapping from the set of locations to higher order words. The transition relations $\xrightarrow[p]{\texttt{std}}$ and $\xrightarrow[p]{\texttt{cert}}$ between process configurations are given in Figure 7; the transition relation $\xrightarrow[p]{\texttt{cert}}$ is used only in the certification phase while $\xrightarrow[p]{\texttt{std}}$ is used to simulate the standard phase of PS 2.0-rlx. A read operation in both phases (standard and certification) is handled by reading a value from a memory type which is on the right of the current pointer of $p$. A write operation, in the standard phase, can result in the insertion, on the right of the current pointer of $p$, of a new memory type at the end of a simple word or as a new simple word. The memory type resulting from a write in the certification phase is only allowed to be inserted at the end of the higher order word or at the reserved slots (using the rule splitting a reservation). Write can also be used to fulfil a promise or to split a promise (i.e., partial fulfilment) during the both phases. Making/canceling a reservation will result in tagging/untagging a memory type at the end of a simple word on the right of the current pointer of $p$. The case of RMW is similar to a read followed by a write operations (whose resulting memory type should be inserted to the right of the read memory type). Finally, a promise can only be made during the standard phase and the resulting memory type will be inserted at the end of a simple word or as a new word on the right of the current pointer of $p$.

$$\frac{\sigma \xrightarrow[p]{\texttt{rd}(x,v)} \sigma', \quad i \geq \mathsf{ptr}(p, \mathbf{HW}(x)), \quad v = \mathbf{HW}(x)[i].value, \qquad \mathbf{HW}' = \mathbf{HW}[x \mapsto \mathsf{mov}(\mathbf{HW}(x), p, i)]}{(\sigma, \mathbf{HW}) \xrightarrow[p]{a} (\sigma', \mathbf{HW}')} \quad \texttt{Read},\ a \in \{\texttt{cert}, \texttt{std}\}$$

$$\frac{\sigma \xrightarrow[p]{\texttt{wt}(x,v)} \sigma', \quad i \geq \mathsf{ptr}(p, \mathbf{HW}(x)), \qquad \mathbf{HW}' = \mathbf{HW}[x \mapsto (\mathbf{HW}(x) \overset{K}{\underset{i}{\hookleftarrow}} (\mathsf{msg}, v, p, \{p\}))]}{(\sigma, \mathbf{HW}) \xrightarrow[p]{\texttt{std}} (\sigma', \mathbf{HW}')} \quad \texttt{Standard write},\ K \in \{N, E\}$$

$$\frac{i \geq \mathsf{ptr}(p, \mathbf{HW}(x)), \quad \mathbf{HW}' = \mathbf{HW}[x \mapsto \mathit{Make}(\mathbf{HW}(x), p, i)]}{(\sigma, \mathbf{HW}) \xrightarrow[p]{\texttt{std}} (\sigma, \mathbf{HW}')} \quad \texttt{Making a reservation}$$

$$\frac{\sigma \xrightarrow[p]{\texttt{U}(x,v_r,w_r)} \sigma', \quad i \geq \mathsf{ptr}(p, \mathbf{HW}(x)), \quad v_r = \mathbf{HW}(x)[i].value, \qquad \mathbf{HW}' = \mathbf{HW}[x \mapsto (\mathbf{HW}(x) \overset{E}{\underset{i}{\hookleftarrow}} (\mathsf{msg}, w_r, p, \{p\}))]}{(\sigma, \mathbf{HW}) \xrightarrow[p]{\texttt{std}} (\sigma', \mathbf{HW}')} \quad \texttt{Standard update}$$

$$\frac{i \geq \mathsf{ptr}(p, \mathbf{HW}(x)), \quad \mathbf{HW}' = \mathbf{HW}[x \mapsto (\mathbf{HW}(x) \overset{E}{\underset{i}{\hookleftarrow}} (\texttt{prm}, v, p, \{\}))]}{(\sigma, \mathbf{HW}) \xrightarrow[p]{\texttt{std}} (\sigma, \mathbf{HW}')} \quad \texttt{Promise}$$

$$\frac{\sigma \xrightarrow[p]{(\mathsf{SC\text{-}fence})} \sigma', \quad i_x = \mathit{max}(\mathsf{ptr}(p, \mathbf{HW}(x)), \mathsf{ptr}(g, \mathbf{HW}(x))), \qquad \mathbf{HW}' = \mathbf{HW}[x \mapsto \mathsf{mov}(\mathbf{HW}(x), p, i_x)]_{x \in \mathsf{Loc}}[x \mapsto \mathsf{mov}(\mathbf{HW}(x), g, i_x)]_{x \in \mathsf{Loc}}}{(\sigma, \mathbf{HW}) \xrightarrow[p]{a} (\sigma', \mathbf{HW}')} \quad \texttt{SC-fence},\ a \in \{\texttt{std}, \texttt{cert}\}$$

Fig. 7: A subset of LoHoW inference rules at the process level.

**Losses in LoHoW.** Let HW and HW′ be two higher order words in $(\Sigma^* \#(\Sigma \cup \Gamma))^+$. Let us assume that $\mathsf{HW} = u_1 \# a_1 u_2 \# a_2 \ldots u_k \# a_k$ and $\mathsf{HW}' = v_1 \# b_1 v_2 \# b_2 \ldots v_m \# b_m$, with $u_i, v_i \in \Sigma^*$ and $a_i, b_j \in \Sigma \cup \Gamma$. We extend the subword relation $\sqsubseteq$ to higher order word as follows: $\mathsf{HW} \sqsubseteq \mathsf{HW}'$ iff there is a strictly increasing function $f : \{1, \ldots, k\} \to \{1, \ldots, m\}$ s.t. (1) $u_i \sqsubseteq v_{f(i)}$ for all $1 \leq i \leq k$, (2) $a_i = b_{f(i)}$, and (3) we have the same number of memory types of the form $(\mathtt{prm}, -, -, -)$ or $(\mathtt{prm}, -, -, -, -)$ in $\mathsf{HW}$ and $\mathsf{HW}'$. The relation $\sqsubseteq$ corresponds to the loss of some special empty memory types and redundant simple words (as explained earlier). The relation $\sqsubseteq$ is extended to mapping from locations to higher order words as follows: $\mathbf{HW} \sqsubseteq \mathbf{HW}'$ iff $\mathbf{HW}(x) \sqsubseteq \mathbf{HW}'(x)$ for all $x \in \mathsf{Loc}$.

**LoHoW states.** A LoHoW state $\mathfrak{st}$ is a tuple $((\mathsf{J}, \mathsf{R}), \mathbf{HW})$ where $\mathsf{J} : \mathcal{P} \mapsto \mathbb{L}$ maps each process $p$ to the label of the next instruction to be executed, $\mathsf{R} : \mathsf{Reg} \to \mathsf{Val}$ maps each register to its current value, and $\mathbf{HW}$ is a mapping from locations to higher order words. The initial LoHoW state $\mathfrak{st}_{\text{init}}$ is defined as $((\mathsf{J}_{\text{init}}, \mathsf{R}_{\text{init}}), \mathbf{HW}_{\text{init}})$ where: (1) $\mathsf{J}_{\text{init}}(p)$ is the label of the initial instruction of $p$; (2) $\mathsf{R}_{\text{init}}(\$r) = 0$ for every $\$r \in \mathsf{Reg}$; and (3) $\mathbf{HW}_{\text{init}}(x) = \mathsf{HW}_x^{\text{init}}$ for all $x \in \mathsf{Loc}$.

For two LoHoW states $\mathfrak{st} = ((\mathsf{J}, \mathsf{R}), \mathbf{HW})$ and $\mathfrak{st}' = ((\mathsf{J}', \mathsf{R}'), \mathbf{HW}')$ and $a \in \{\mathtt{std}, \mathtt{cert}\}$, we write $\mathfrak{st} \xrightarrow[p]{a} \mathfrak{st}'$ iff one of the following cases holds: (1) $((\mathsf{J}(p), \mathsf{R}), \mathbf{HW}) \xrightarrow[p]{a} ((\mathsf{J}'(p), \mathsf{R}'), \mathbf{HW}')$ and $\mathsf{J}(p') = \mathsf{J}'(p')$ for all $p' \neq p$, or (2) $(\mathsf{J}, \mathsf{R}) = (\mathsf{J}', \mathsf{R}')$ and $\mathbf{HW} \sqsubseteq \mathbf{HW}'$.

**Two phases LoHoW states**. A two-phases state of LoHoW is $\mathcal{S} = (\pi, p, \mathfrak{st}_{\mathtt{std}}, \mathfrak{st}_{\mathtt{cert}})$ where $\pi \in \{\mathtt{cert}, \mathtt{std}\}$ is a flag describing whether the LoHoW is in "standard" phase or "certification" phase, $p$ is the process which evolves in one of these phases, while $\mathfrak{st}_{\mathtt{std}}$, $\mathfrak{st}_{\mathtt{cert}}$ are two LoHoW states (one for each phase). When the LoHoW is in the standard phase, then $\mathfrak{st}_{\mathtt{std}}$ evolves, and when the LoHoW is in certification phase, $\mathfrak{st}_{\mathtt{cert}}$ evolves. A two-phases LoHoW state is said to be initial if it is of the form $(\mathtt{std}, p, \mathfrak{st}_{\text{init}}, \mathfrak{st}_{\text{init}})$, where $p \in \mathcal{P}$ is any process. The transition relation $\to$ between two-phases LoHoW states is defined as follows: Given $\mathcal{S} = (\pi, p, \mathfrak{st}_{\mathtt{std}}, \mathfrak{st}_{\mathtt{cert}})$ and $\mathcal{S}' = (\pi', p', \mathfrak{st}'_{\mathtt{std}}, \mathfrak{st}'_{\mathtt{cert}})$, we have $\mathcal{S} \to \mathcal{S}'$ iff one of the following cases holds:

- **During the standard phase.** $\pi = \pi' = \mathtt{std}$, $p = p'$, $\mathfrak{st}_{\mathtt{cert}} = \mathfrak{st}'_{\mathtt{cert}}$ and $\mathfrak{st}_{\mathtt{std}} \xrightarrow[p]{\mathtt{std}} \mathfrak{st}'_{\mathtt{std}}$. This corresponds to simulating a standard step of process $p$.
- **During the certification phase.** $\pi = \pi' = \mathtt{cert}$, $p = p'$, $\mathfrak{st}_{\mathtt{std}} = \mathfrak{st}'_{\mathtt{std}}$ and $\mathfrak{st}_{\mathtt{cert}} \xrightarrow[p]{\mathtt{cert}} \mathfrak{st}'_{\mathtt{cert}}$. This simulates a certification step of process $p$.
- **From the standard phase to the certification phase.** $\pi = \mathtt{std}$, $\pi' = \mathtt{cert}$, $p = p'$, $\mathfrak{st}_{\mathtt{std}} = \mathfrak{st}'_{\mathtt{std}} = ((\mathsf{J}, \mathsf{R}), \mathbf{HW})$, and $\mathfrak{st}'_{\mathtt{cert}}$ is of the form $((\mathsf{J}, \mathsf{R}), \mathbf{HW}')$ where for every $x \in \mathsf{Loc}$, $\mathbf{HW}'(x) = \mathbf{HW}(x)\#(\mathsf{msg}, v, q, \{\})$ if $\mathbf{HW}(x)$ is of the form $w \cdot \#(-, v, -, -, q)$ with $q \neq p$, and $\mathbf{HW}'(x) = \mathbf{HW}(x)$ otherwise. This corresponds to the copying of the standard LoHoW state to the certification LoHoW state in order to check if the set of promises made by the process $p$ can be fulfilled. This transition rule can be implemented by a sequence of transitions which copies one symbol at a time, from $\mathbf{HW}$ to $\mathbf{HW}'$.

– **From the certification phase to standard phase.** $\pi = \texttt{cert}$, $\pi' = \texttt{std}$, $\mathfrak{st}_{\texttt{std}} = \mathfrak{st}'_{\texttt{std}}$, $\mathfrak{st}_{\texttt{cert}} = \mathfrak{st}'_{\texttt{cert}}$, and $\mathfrak{st}_{\texttt{cert}}$ is of the form $((\mathsf{J}, \mathsf{R}), \mathbf{HW})$ with $\mathbf{HW}(x)$ does not contain any memory type of form $(\texttt{prm}, -, p, -, \underline{?})$ for all $x \in \mathsf{Loc}$ (i.e., all promises made by $p$ are fulfilled).

**The Reachability Problem in** LoHoW. Given an instruction label function $J : \mathcal{P} \to \mathbb{L}$ that maps each $p \in \mathcal{P}$ to a label in $\mathbb{L}_p$, the *reachability* problem in LoHoW asks whether there exists a two phases LoHoW state $\mathcal{S}$ of the form $(\texttt{std}, -, ((J, R), \mathbf{HW}), ((J', R'), \mathbf{HW}'))$ s.t. (1) $\mathbf{HW}(x)$ and $\mathbf{HW}'(x)$ do not contain any memory type of the form $(\texttt{prm}, -, p, -, \underline{?})$ for all $x \in \mathsf{Loc}$, and (2) $\mathcal{S}$ is reachable in LoHoW (i.e., $\mathcal{S}_0\ [\to]^*\ \mathcal{S}'$ where $\mathcal{S}_0$ is an initial two-phases LoHoW states). A positive answer to this problem means $J$ is reachable in *Prog* in LoHoW. The following theorem states the equivalence between LoHoW and PS 2.0-rlx in terms of reachable instruction label functions.

**Theorem 2.** *An instruction label function $J$ is reachable in a program Prog in* LoHoW *iff $J$ is reachable in Prog in PS 2.0-rlx.*

### 5.2 Decidability of LoHoW with Bounded Promises

The equivalence of the reachability in LoHoW and PS 2.0-rlx, coupled with Theorem 1 shows that reachability is undecidable in LoHoW. To recover decidability, we look at LoHoW with only bounded number of the promise memory type in any higher order word. Let K-LoHoW denote LoHoW with a number of promises bounded by $K$. (Observe that K-LoHoW corresponds to bdPS 2.0-rlx.)

**Theorem 3.** *The reachability problem is decidable for $K$-*LoHoW.

As a corollary of Theorem 3, the decidability of reachability follows for bdPS 2.0-rlx. The proof makes use of the framework of *Well-Structured Transition Systems* (WSTS) [7,13]. Next, we state that the reachability problem for K-LoHoW (even for $K = 0$) is highly non-trivial (i.e., non-primitive recursive). The proof is done by reduction from the reachability problem for lossy channel systems, in a similar to the case of TSO [8] where we insert SC-fence instructions everywhere in the process that simulates the lossy channel process (in order to ensure that no promises can be made by that process).

**Theorem 4.** *The reachability problem for $K$-*LoHoW *is non-primitive recursive.*

## 6 Source to Source Translation

In this section, we propose an algorithmic approach for state reachability in concurrent programs under PS 2.0. We first recall the notion of view altering reads [1], and that of bounded contexts in SC [29].

*View Altering Reads.* A read from the memory is view altering if it changes the view of the process reading it. This means that the view in the message being read from was greater than the process view on some variable. The message which is read from in turn is called a view altering message. A run in which the total number of view altering reads (across all threads) is bounded (by some parameter) is called a view-bounded run. The underapproximate analysis for PS 2.0-ra without promises and reservations [1] considered view bounded runs.

*Essential Events.* An essential event in a run $\rho$ of a program under PS 2.0 is either a promise, a reservation or a view altering read by some process in the run.

*Bounded Context.* A context is an uninterrupted sequence of actions by a single process. In a run having $K$ contexts, the execution switches from one process to another $K-1$ times. A $K$ bounded context run is one where the number of context switches are bounded by $K \in \mathbb{N}$. The $K$ bounded context reachability problem in SC checks for the existence of a $K$ bounded context run reaching some chosen instruction. Now we define the notion of bounding for PS 2.0.

**The Bounded Consistent Reachability Problem**. A run $\rho$ of a concurrent program under PS 2.0, $\mathcal{MS}_0 \, [\xrightarrow[p_{i_1}]{}]^* \, \mathcal{MS}_1 \, [\xrightarrow[p_{i_2}]{}]^* \, \mathcal{MS}_2 \, [\xrightarrow[p_{i_3}]{}]^* \ldots [\xrightarrow[p_{i_n}]{}]^* \, \mathcal{MS}_n$ is called $K$ *bounded* iff the number of essential events in $\rho$ is $\leq K$. The $K$ bounded reachability problem for PS 2.0 checks for the existence of a run $\rho$ of *Prog* which is $K$-bounded. Assuming *Prog* has $n$ processes, we propose an algorithm that reduces the $K$ bounded reachability problem to a $K+n$ bounded context reachability problem of a program $[\![Prog]\!]$ under SC.

**Translation Overview**. We now provide a brief overview of the data structures and procedures utilized in our translation; the full details and correctness are in [5]. Let *Prog* be a concurrent program under PS 2.0 with set of processes $\mathcal{P}$ and locations Loc. Our algorithm relies on a source to source translation of *Prog* to a bounded context SC program $[\![Prog]\!]$, as shown in Figure 8 and operates on the same data domain (need not be finite). The translation (i) adds a new process (Main) that initializes the global variables of $[\![Prog]\!]$, (2) for each process $p \in \mathcal{P}$ adds local variables, which are initialized by the function InitProc.

$$
\begin{aligned}
[\![Prog]\!] &:= (\langle \text{global vars}\rangle; \langle \textsc{Main}\rangle; ([\![\texttt{proc}\ p\ \texttt{reg}\ \$r^* i^*]\!])^* \\
[\![\texttt{proc}\ p\ \texttt{reg}\ \$r^*\ i^*]\!] &:= \texttt{proc}\ p\ \texttt{reg}\ \$r^* \\
&\qquad \langle \text{local vars}\rangle \langle \textsc{InitProc}\rangle \langle \text{CSO}\rangle^{p,\lambda_0} ([\![i]\!]^p)^* \\
[\![\lambda : i]\!]^p &:= \lambda : \langle \text{CSI}\rangle; [\![s]\!]^p; \langle \text{CSO}\rangle^{p,\lambda} \\
[\![\texttt{if}\ exp\ \texttt{then}\ i^*\ \texttt{else}\ i^*]\!]^p &:= \texttt{if}\ exp\ \texttt{then}\ ([\![i]\!]^p)^*\ \texttt{else}([\![i]\!]^p)^* \\
[\![\texttt{while}\ exp\ \texttt{do}\ i^*]\!]^p &:= \texttt{while}\ exp\ \texttt{do}\ ([\![i]\!]^p)^* \\
[\![\texttt{assume}(exp)]\!]^p &:= \texttt{assume}(exp) \\
[\![\$r = exp]\!]^p &:= \$r = exp \\
[\![x = \$r]\!]^p_{o \in \{\texttt{rlx},\texttt{ra}\}} &:= \ \text{see write Pseudocode} \\
[\![\$r = x]\!]^p_{o \in \{\texttt{rlx},\texttt{ra}\}} &:= \ \text{see read Pseudocode}
\end{aligned}
$$

Fig. 8: Source-to-source translation map

This is followed by the code block $\langle CSO\rangle^{p,\lambda_0}$ (Context Switch Out) that optionally enables the process to switch out of context. For each $\lambda$ labeled instruction $i$ in $p$, the map $[\![\lambda : i]\!]^p$ transforms it into a sequence of instructions as follows : the code block $\langle CSI \rangle$ (Context Switch In) checks if the process is active in the current context; then it transforms each statement $s$ of instruction $i$ into a sequence of instructions following the map $[\![s]\!]^p$, and finally executes the code block $\langle CSO \rangle^{p,\lambda}$. $\langle CSO \rangle^{p,\lambda}$ facilitates two things: when the process is at an instruction label $\lambda$, (1) allows $p$ to make promises/reservations after $\lambda$, s.t. the control is back at $\lambda$ after certification; (2) it ensures that the machine state is consistent when $p$ switches out of context. Translation of `assume`, `if` and `while` statements keep the same statement. Translation of read and write statements are described later. Translation of RMW statements are omitted for ease of presentation.

The set of promises a process makes has to be constrained with respect to the set of promises that it can certify To address this, in the translation, processes run in two modes : a 'normal' mode and a 'check' (*consistency check*) mode. In the normal mode, a process does not make any promises or reservations. In the check mode, the process may make promises and reservations and subsequently certify them before switching out of context. In any context, a process first enters the normal mode, and then, before exiting the context it enters the check mode. The check mode is used by the process to (1) make new promises/reservations and (2) certify consistency of the machine state. We also add an optional parameter, called *certification depth* (`certDepth`), which constrains the number of steps a process may take in the check mode to certify its promises. Figure 9 shows the structure of a translated run under SC.

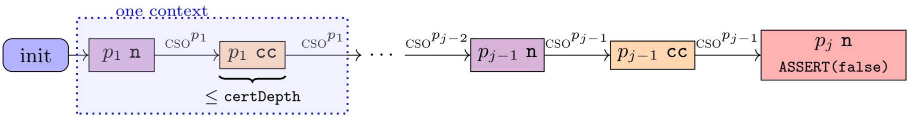

Fig. 9: Control flow: In each context, a process runs first in normal mode `n` and then in consistency check mode `cc`. The transitions between these modes is facilitated by the CSO code block of the respective process. We check assertion failures for $K + n$ context-bounded executions ($j \leq K + n$).

To reduce the PS 2.0 run into a bounded context SC run, we use the bound on the number of essential events. From the run $\rho$ in PS 2.0, we construct a $K$ bounded run $\rho'$ in PS 2.0 where the processes run in the order of generation of essential events. So, the process which generates the first essential event is run first, till that event happens, then the second process which generates the second essential event is run, and so on. This continues till $K + n$ contexts : the $K$ bounds the number of essential events, and the $n$ is to ensure all processes are run to completion. The bound on the number of essential events gives a bound on the number of timestamps that need to be maintained. As observed in [1], each view altering read requires two timestamps; additionally, each promise/reservation requires one timestamp. Since we have $K$ such essential events, $2K$ time stamps suffice. We choose $\mathsf{Time} = \{0, 1, 2, \ldots, 2K\}$ as the set of timestamps. Now we briefly give a high level overview of the translation.

**Data Structures**. The `message` data structure represents a message generated as a write or a promise and has 4 fields (i) *var*, the address of the memory location written to; (ii) the timestamp $t$ in the view associated with the message; (iii) $v$, the value written; and (iv) *flag*, that keeps track of whether it is a message or a promise; and, in case of a promise, which process it belongs to. The View data structure stores, for each memory location $x$, (i) a timestamp $t \in \mathsf{Time}$, (ii) a value $v$ written to $x$, (iii) a Boolean $l \in \{\mathtt{true}, \mathtt{false}\}$ representing whether $t$ is an *exact* timestamp (which can be used for essential events) or an *abstract* timestamp (which corresponds to non-essential events).

**Global Variables**. The `Memory` is an array of size $K$ holding elements of type `message` . This array is populated with the view altering messages, promises and reservations generated by the program. We maintain counters for (1) the number of elements in `Memory` ; (2) the number of context switches that have occurred; and (3) the number of essential events that have occurred.

**Local Variables**. In addition to its local registers, each process has local variables including (1) a local variable `view` which stores a local instance of the view function (this is of type View), (2) a flag denoting whether the process is running in the current context, and (3) a flag *checkMode* denoting whether the process is in the certification phase. We implement the certification phase as a function call, and hence store the process state and return address, while entering it.

### 6.1 Translation Maps

In what follows we illustrate how the translation simulates a run under PS 2.0. At the outset, recall that each process alternates, in its execution, between two modes: a *normal* mode (`n` in Figure 9) at the beginning of each context and the *check* mode at the end of the current context (`cc` in Figure 9), where it may make new promises and certify them before switching out of context.

**Context Switch Out ($CSO^{p,\lambda}$).** We describe the CSO module; Algorithm 1 of Figure 10 provides its pseudocode. CSO$^{p,\lambda}$ is placed after each instruction $\lambda$ in the original program and serves as an entry and exit point for the consistency check phase of the process. When in normal mode (`n`) after some instruction $\lambda$, CSO non-deterministically guesses whether the process should exit the context at this point, and sets the *checkMode* flag to true and subsequently, saves its local state and the return address (to mark where to resume execution from, in the next context). The process then continues its execution in the consistency check mode (`cc`) from the current instruction label ($\lambda$) itself. Now the process may generate new promises (see Algorithm 1 of Figure 10) and certify these as well as earlier made promises. In order to conclude the check mode phase, the process will enter the CSO block at some different instruction label $\lambda'$. Now since the *checkMode* flag is true, the process enters the else branch, verifies that there are no outstanding promises of $p$ to be certified. Since the promises are not yet fulfilled, when $p$ switches out of context, it has to mark all its promises uncertified. When the context is back to $p$ again, this will be used to fulfil the promises or to certify them again before the context switches out of $p$ again.

Then it exits the check mode phase, setting *checkMode* to false. Finally it loads the saved state, and returns to the instruction label $\lambda$ (where it entered check mode) and exits the context. Another process may now resume execution.

**Algorithm 1:** CSO

```
/* nondeterministically enter
   check mode and exit context   */
if nondet() then
    if ¬checkMode then
        /* enter consistency check
           */
        if not in context then
            enter context
        end
        checkMode ← true
        save localstate
        returnAddr ← λ
    else
        /* consistency check
           successful!           */
        ensure all promises certified

/* for next context      */
        mark all promises
          uncertified
        checkMode ← false
        load localstate
        goto returnAddr
        exit context
    end
end
```

**Algorithm 2:** Write

```
update localstate with write
if nondet() then
            /* (i) no fresh timestamp */
    if checkMode then
        /* write is not a promise    */
        certify message with reservation
          or splitting
else if nondet() then /* (ii) new ts */
    generate a view; generate a message
    if checkMode then
        insert message into Memory as
          Promise and certify
    else
        insert message into Memory as
          concrete message
    end
else      /* (iii) fulfill old promise */
    get Promise from Memory
    check variable, value and view match
    if checkMode then
        mark message as certified
    else
        mark message as fulfilled
    end
    replace message into Memory
end
```

Fig. 10: Algorithms for CSO and Write

**Write Statements**. The translation of a write instruction $[\![x := \$r]\!]_o$, where $o \in \{\texttt{rlx}, \texttt{ra}\}$ of a process $p$ is given in Algorithm 2 of Figure 10. This is the general pseudo code for both kinds of memory accesses, with specific details pertaining to the particular access mode omitted. Let us first consider execution in the normal mode (i.e., *checkMode* is false). First, the process updates its local state with the value that it will write. Then, the process non-deterministically chooses one of three possibilities for the write, it either (i) does not assign a fresh timestamp (non-essential event), (ii) assigns a fresh timestamp and adds it to memory, or (iii) fulfils some outstanding promise.

Let us now consider a write executing when *checkMode* is true, and highlight differences with the normal mode. In case (i), non essential events exclude promises and reservations. Then, while in certification phase, since we use a capped memory, the process can make a write if either (1) the write interval can be generated through splitting insertion or (2) the write can be certified with the help of a reservation. Basically the writes we make either split an existing interval (and add this to the left of a promise), or forms a part of a reservation. Thus, the time stamp of a neighbour is used. In case (ii) when a fresh time stamp is used, the write is made as a promise, and then certified before switching out of context. The analogue of case (iii) is the certification of promises for the current context; promise fulfilment happens only in the normal mode. To help a process decide the value of a promise, we use the fact that CBMC allows us to assign a non-deterministic value of a variable. On top of that, we have implemented an optimization that checks the set of possible values to be written in the future.

**Read Statements.** The translation of a read instruction $[\![\$r := x]\!]_o$, $o \in \{\texttt{rlx}, \texttt{ra}\}$ of process $p$ is given in Algorithm 3 of Figure 11.

The process first guesses, whether it will read from a view altering message in the memory of from its local view. If it is the latter, the process must first verify whether it can read from the local view ; for instance, reading from the local view may not be possible after execution of a `fence` instruction when the timestamp of a variable $x$ gets incremented from the local view $t$ to $t' > t$. In the case of a view altering read, we first check that we have not reached the context switching/essential event bound. Then the new `message` is fetched from `Memory` and we check the view (timestamps) in the acquired `message` satisfy the conditions imposed by the access type $\in \{\texttt{ra}, \texttt{rlx}\}$. Finally, the process updates its view with that of the new message and increments the counters for the context switches and the essential events. Theorem 5 proves the correctness of our translation.

**Algorithm 3:** `Read`

```
if nondet() then /* local read */
    check local state is valid
    update local state with read
else                    /* nonlocal (view-switching) read */
    check if local state allows read and get message from
    Memory. check new message
    satisfies conditions for read update local state
end
```

Fig. 11: Algorithm for `Read`

**Theorem 5.** *Given a program Prog under PS 2.0, and $K \in \mathbb{N}$, the source to source translation constructs a program $[\![prog]\!]$ whose size is polynomial in Prog and $K$ such that, there is a $K$-bounded run of Prog under PS 2.0 reaching a set of instruction labels, if and only if there is a $K{+}n$-bounded context run of $[\![prog]\!]$ under SC that reaches the same set of instruction labels.*

## 7 Implementation and Experimental Results

In order to check the efficiency of the source-to-source translation, we implement a prototype tool, PS2SC which is the first tool to handle PS 2.0. PS2SC takes as input a C program and a bound $K$ and translates it to a program $Prog'$ to be run under SC. We use CBMC v5.10 as the backend verifier for $Prog'$. CBMC takes as input $L$, the loop unrolling parameter for bounded model checking of $Prog'$. If PS2SC returns *unsafe*, then the program has an unsafe execution. Conversely, if it returns *safe* then none of the executions within the subset violate any assertion. $K$ may be iteratively incremented to increase the number of executions explored. PS2SC has a functionality of *partial-promises* allowing subsets of processes to promise, providing an effective under-approximation technique.

We now report the results of experiments we have performed with PS2SC. We have two objectives: (1) studying the performance of PS2SC on thin-air litmus tests and benchmarks utilizing promises, and (2) comparing PS2SC with other model checkers when operating in the promise-free mode. In the first case we show that PS2SC is able to uncover bugs in litmus tests and examples with few reads and writes to the shared memory. When this interaction and subsequent non-determinism of PS 2.0 increases, we also enable *partial promises*. For the second case we compare PS2SC with three model checkers CDSChecker [25], GenMC [18] and Rcmc [17] that support the promise-free subset of PS 2.0. Our observations highlight the ability to detect hard to find bugs with small $K$ for unsafe benchmarks. We do not consider compilation time for any tool while reporting the results. For PS2SC, the time reported is the time taken by the CBMC backend for analysis. The timeout used is 1hr for all benchmarks. All experiments are conducted on a machine with 3.00 GHz Intel Core i5-3330 CPU and 8GB RAM running an Ubuntu-16 64-bit operating system. We denote timeout by 'TO', and memory limit exceeded by 'MLE'.

**Benchmarks Utilizing Promises.** In the following, we report the performance of PS2SC on litmus tests and parametrized tests.

*Litmus Tests.* We test PS2SC on litmus-tests adapted from [16,22,11,23]. These examples are small programs that serve as barebones thin-air tests for the C11 memory model. Consistency tests based on the Java Memory Model are proposed in [23], which were experimented on by [27] with their MRDer tool. Like MRDer, PS2SC is able to verify most of these tests within 1 minute which shows its ability to handle typical programming idioms of PS 2.0 (see Table 1).

| **testcase** | $K$ | PS2SC |
|---|---|---|
| ARM_weak | 4 | 0.765s |
| Upd-Stuck | 4 | 1.252s |
| split | 4 | 25.737s |
| LBd | 3 | 1.481s |
| LBfd | 3 | 1.512s |
| CYC | 5 | 1.967s |
| Coh-CYC | 5 | 42.67s |
| Pugh2 | 3 | 13.725s |
| Pugh3 | 3 | 12.920s |
| Pugh8 | 3 | 1.67s |
| Pugh5 | 5 | 4.811s |
| Pugh10 | 5 | 3.868s |
| Pugh13 | 5 | 3.345s |

Table 1: Litmus Tests

*Parameterized Tests.* In Table 2, we consider unsafe examples adapted from the `Fibonacci`-based benchmarks of SV-COMP 2019 [10]. In these examples a process is required to generate a promise (speculative write) with value as the $i^{th}$ fibonacci number. This promise is certified using *process-local* reads. Thus though the parameter $i$ increases the interaction of the promising process with the memory remains constant. The **CAS** variant requires the process to make use of reservations. We note that PS2SC uncovers the bugs effectively in these cases. In cases where promise-certificate requires reads from external processes, the amount of shared-memory interaction increases with $i$. In this case, we use *partial promises*.

| **testcase** | $K$ | PS2SC |
|---|---|---|
| fib_local_3 | 4 | 0.742s |
| fib_local_4 | 4 | 0.761s |
| fib_local_cas_3 | 4 | 1.132s |
| fib_local_cas_4 | 4 | 1.147s |
| **testcase** | $K$ | PS2SC[1p] |
| fib_global_2 | 4 | 55.972s |
| fib_global_3 | 4 | 2m4s |
| fib_global_4 | 4 | 4m20s |
| exp_global_1 | 4 | 19m37s |
| exp_global_2 | 4 | 41m12s |

Table 2: Above: testcases with local reads, Below: global reads

*How to recover tractable analysis?* We note that though the above example consists of several processes interacting with the memory, the bug can be uncovered even if only a *single* process is allowed to make promising writes. We run PS2SC in the partial-promises mode. We considered the case where only a single process generates promises, and PS2SC was able to uncover the bug. The results obtained are in Table 2, where PS2SC[1p] denotes that only one process is permitted to perform promises. We then repeat our experiments on other unsafe benchmarks - including `ExponentialBug` from Fig. 2 of [15] - and have similar observations. To summarize, we note that the huge non-determinism of PS 2.0 can be fought by using the modular approach of partial-promises.

**Comparing with Other Tools.** In this section, we compare performance of PS2SC in promise-free mode with CDSChecker [25], GenMC [18] and Rcmc [17] (which do not support promises). The main objective of this section is to provide evidence for the practicability of the essential-event-bounding technique. The results of this section indicate that the source-to-source translation with $K$-essential-event bounding is effective at uncovering hard to find bugs in non-trivial programs. Additionally, we observe that in most examples considered, we had $K \leq 10$. We provide here a subset of the experimental results and the remaining in the full version of the paper [5]. In the tables that follow we provide the value of $K$ (for PS2SC) and the value of $L$ (loop-unrolling bound) for all tools.

*Parameterized Benchmarks.* In Table 3, we experiment on two parametrized benchmarks: `ExponentialBug` (Fig. 2 of [15]) and `Fibonacci` (from SV-COMP 2019). In `ExponentialBug`($N$) $N$ is the number writes made to a variable by a process. We note that in `ExponentialBug`($N$) the number of executions grows as $N!$, while the processes have to follow a specific interleaving to uncover the hard to find bug. In `Fibonacci`($N$), two processes compute the value of the $n^{th}$ fibonacci number in a distributed fashion.

| **benchmark** | $L$ | $K$ | PS2SC | **CDSC** | **GenMC** | **RCMC** |
|---|---|---|---|---|---|---|
| exponential_25_unsafe | 25 | 10 | 3.532s | 7.239s | 3.736s | TO |
| exponential_50_unsafe | 50 | 10 | 6.128s | 36.361s | 39.920s | TO |
| fibonacci_3_unsafe | 3 | 20 | 9.392s | 46m8s | 0.462s | 0.544s |
| fibonacci_4_unsafe | 4 | 20 | 34.019s | TO | 12.437s | 18.953s |

Table 3: Parameterized benchmarks

*Concurrent data structures based benchmarks.* In Table 4, we consider benchmarks based on concurrent data structures. The first of these is a concurrent locking algorithm originating from [14]. The second, `LinuxLocks(N)` is adapted from evaluations of CDSChecker [25]. We note that if not completely fenced, it is unsafe. We fence all but one lock access. Both these results show the ability of our tool to uncover bugs with a small value of $K$.

| **benchmark** | $L$ | $K$ | PS2SC | **CDSC** | **GenMC** | **RCMC** |
|---|---|---|---|---|---|---|
| hehner2_unsafe | 4 | 5 | 7.207s | 0.033s | 0.094s | 0.087s |
| hehner3_unsafe | 4 | 5 | 28.345s | 0.036s | 2m53s | 1m13s |
| linuxlocks2_unsafe | 2 | 4 | 0.547s | 0.032s | 0.073s | 0.078s |
| linuxlocks3_unsafe | 2 | 4 | 1.031s | 0.031s | 0.083s | 0.081s |

Table 4: Concurrent data structures

*Variations of mutual exclusion protocols.* We consider variants of mutual exclusion protocols from SV-COMP 2019. The fully fenced versions of the protocols are *safe*. We modify these protocols by introducing bugs and comparing the performance of PS2SC for bug detection with the other tools. These benchmarks are parameterized by the number of processes. In Table 5, we unfence a single process of the Peterson and Szymanski protocols making them *unsafe*. These are benchmarks `petersonU(i)` and `szymanskiU(i)` where `i` is the number of processes.

In `petersonB(i)`, we keep all processes fenced but introduce a bug into the critical section of a process (write a value to a shared variable and read a different value from it). We note that the other tools do not scale, while PS2SC is able to detect the bug within one minute, showing that essential event-bounding is an effective under-approximation technique for bug-finding.

| **benchmark** | $L$ | $K$ | PS2SC | **CDSChecker** | **GenMC** | **RCMC** |
|---|---|---|---|---|---|---|
| petersonU(4) | 1 | 6 | 1.408s | 0.039s | TO | 9.129s |
| petersonU(8) | 1 | 6 | 47.786s | TO | TO | TO |
| szymanskiU(4) | 1 | 2 | 1.015s | 0.043s | MLE | TO |
| szymanskiU(8) | 1 | 2 | 6.176s | TO | TO | TO |
| petersonB(3) | 1 | 2 | 0.487s | 0.053s | 0.083s | 0.087s |
| petersonB(5) | 1 | 2 | 2.713s | TO | TO | TO |
| petersonB(7) | 1 | 2 | 11.008s | TO | TO | TO |

Table 5: Mutual exclusion benchmarks with a single unfenced process

**Remark.** Through all these experiments, we observe that SMC tools and our tool try to tackle the same problem by using orthogonal approaches to finding bugs. Hence, through the experiments above we are not trying to pitch one approach against the other, but rather trying to highlight the differences in their features. We have exhibited examples where our tool is able to uncover hard-to-find bugs faster than the others with relatively small values of $K$.

## 8 Related Work and Conclusion

Most of the existing verification work for C/C++ concurrency models concern the development of stateless model checking coupled with dynamic partial order reduction (e.g., [6,17,18,26,25]) and do not handle the promising semantics. Context-bounding has been proposed in [29] for programs running under SC. This work has been extended in different directions and has led to efficient and scalable techniques for the analysis of concurrent programs (see e.g., [24,21,33,32,12,34]). In the context of weak memory models, context-bounded analyses have been proposed for TSO/PSO [9,31] and POWER [3].

The decidability of the verification problems for programs running under weak memory models has been addressed for TSO [8], RA [1], SRA [19], and POWER [2]. We believe that our proof techniques can be easily adapted to work with different variants of the promising semantics [16] (see [4]). For instance, in the code-to-code translation, the mechanism for making and certifying promises and reservations is isolated in one module, which can be easily changed to cover different variants of the promising semantics. Furthermore, the undecidability proof still goes through for [16]. Moreover, providing a tool for the verification of (among other things) litmus tests, will provide a valuable environment which can be used in further improvements of the promising semantics. To the best of our knowledge, this the first time that this problem is investigated for PS 2.0-rlx and PS2SC is the first tool for automated verification of programs under PS 2.0. Finally, studying the decidability problem for related models that solve the thin-air problem (e.g., Paviotti et al. [27]) is interesting and kept as future work.

## References

[1] Abdulla, P.A., Arora, J., Atig, M.F., Krishna, S.N.: Verification of programs under the release-acquire semantics. In: McKinley, K.S., Fisher, K. (eds.) Proceedings of the 40th ACM SIGPLAN Conference on Programming Language Design and Implementation, PLDI 2019, Phoenix, AZ, USA, June 22-26, 2019. pp. 1117–1132. ACM (2019)

[2] Abdulla, P.A., Atig, M.F., Bouajjani, A., Derevenetc, E., Leonardsson, C., Meyer, R.: Safety verification under power. In: NETYS 2020. Lecture Notes in Computer Science, Springer (2020), to appear

[3] Abdulla, P.A., Atig, M.F., Bouajjani, A., Ngo, T.P.: Context-bounded analysis for POWER. In: Legay, A., Margaria, T. (eds.) Tools and Algorithms for the Construction and Analysis of Systems - 23rd International Conference, TACAS 2017, Held as Part of the European Joint Conferences on Theory and Practice of Software, ETAPS 2017, Uppsala, Sweden, April 22-29, 2017, Proceedings, Part II. Lecture Notes in Computer Science, vol. 10206, pp. 56–74. Springer (2017)

[4] Abdulla, P.A., Atig, M.F., Godbole, A., Krishna, S.N., Vafeiadis, V.: Verification of c11 programs with relaxed accesses (2019), https://www.cse.iitb.ac.in/~krishnas/ps1.pdf

[5] Abdulla, P.A., Atig, M.F., Godbole, A., Krishna, S.N., Vafeiadis, V.: The decidability of verification under promising 2.0. CoRR **abs/2007.09944** (2020), https://arxiv.org/abs/2007.09944

[6] Abdulla, P.A., Atig, M.F., Jonsson, B., Ngo, T.P.: Optimal stateless model checking under the release-acquire semantics. Proc. ACM Program. Lang. **2**(OOPSLA), 135:1–135:29 (2018)

[7] Abdulla, P.A., Jonsson, B.: Verifying programs with unreliable channels. Inf. Comput. **127**(2), 91–101 (1996)

[8] Atig, M.F., Bouajjani, A., Burckhardt, S., Musuvathi, M.: On the verification problem for weak memory models. In: Hermenegildo, M.V., Palsberg, J. (eds.) Proceedings of the 37th ACM SIGPLAN-SIGACT Symposium on Principles of Programming Languages, POPL 2010, Madrid, Spain, January 17-23, 2010. pp. 7–18. ACM (2010)

[9] Atig, M.F., Bouajjani, A., Parlato, G.: Getting rid of store-buffers in TSO analysis. In: Gopalakrishnan, G., Qadeer, S. (eds.) Computer Aided Verification - 23rd International Conference, CAV 2011, Snowbird, UT, USA, July 14-20, 2011. Proceedings. Lecture Notes in Computer Science, vol. 6806, pp. 99–115. Springer (2011)

[10] Beyer, D.: Automatic verification of C and java programs: SV-COMP 2019. In: Beyer, D., Huisman, M., Kordon, F., Steffen, B. (eds.) Tools and Algorithms for the Construction and Analysis of Systems - 25 Years of TACAS: TOOLympics, Held as Part of ETAPS 2019, Prague, Czech Republic, April 6-11, 2019, Proceedings, Part III. Lecture Notes in Computer Science, vol. 11429, pp. 133–155. Springer (2019)

[11] Chakraborty, S., Vafeiadis, V.: Grounding thin-air reads with event structures. Proc. ACM Program. Lang. **3**(POPL), 70:1–70:28 (2019)

[12] Emmi, M., Qadeer, S., Rakamaric, Z.: Delay-bounded scheduling. In: Ball, T., Sagiv, M. (eds.) Proceedings of the 38th ACM SIGPLAN-SIGACT Symposium on Principles of Programming Languages, POPL 2011, Austin, TX, USA, January 26-28, 2011. pp. 411–422. ACM (2011)

[13] Finkel, A., Schnoebelen, P.: Well-structured transition systems everywhere! Theor. Comput. Sci. **256**(1-2), 63–92 (2001)

[14] Hehner, E.C.R., Shyamasundar, R.K.: An implementation of P and V. Inf. Process. Lett. **12**(4), 196–198 (1981)

[15] Huang, J.: Stateless model checking concurrent programs with maximal causality reduction. In: Grove, D., Blackburn, S. (eds.) Proceedings of the 36th ACM SIGPLAN Conference on Programming Language Design and Implementation, Portland, OR, USA, June 15-17, 2015. pp. 165–174. ACM (2015)

[16] Kang, J., Hur, C., Lahav, O., Vafeiadis, V., Dreyer, D.: A promising semantics for relaxed-memory concurrency. In: Castagna, G., Gordon, A.D. (eds.) Proceedings of the 44th ACM SIGPLAN Symposium on Principles of Programming Languages, POPL 2017, Paris, France, January 18-20, 2017. pp. 175–189. ACM (2017)

[17] Kokologiannakis, M., Lahav, O., Sagonas, K., Vafeiadis, V.: Effective stateless model checking for C/C++ concurrency. Proc. ACM Program. Lang. **2**(POPL), 17:1–17:32 (2018)

[18] Kokologiannakis, M., Raad, A., Vafeiadis, V.: Model checking for weakly consistent libraries. In: McKinley, K.S., Fisher, K. (eds.) Proceedings of the 40th ACM SIGPLAN Conference on Programming Language Design and Implementation, PLDI 2019, Phoenix, AZ, USA, June 22-26, 2019. pp. 96–110. ACM (2019)

[19] Lahav, O., Boker, U.: Decidable verification under a causally consistent shared memory. In: Donaldson, A.F., Torlak, E. (eds.) Proceedings of the 41st ACM SIGPLAN International Conference on Programming Language Design and Implementation, PLDI 2020, London, UK, June 15-20, 2020. pp. 211–226. ACM (2020)

[20] Lahav, O., Vafeiadis, V., Kang, J., Hur, C., Dreyer, D.: Repairing sequential consistency in C/C++11. In: Cohen, A., Vechev, M.T. (eds.) Proceedings of the 38th ACM SIGPLAN Conference on Programming Language Design and Implementation, PLDI 2017, Barcelona, Spain, June 18-23, 2017. pp. 618–632. ACM (2017)

[21] Lal, A., Reps, T.W.: Reducing concurrent analysis under a context bound to sequential analysis. Formal Methods Syst. Des. **35**(1), 73–97 (2009)

[22] Lee, S., Cho, M., Podkopaev, A., Chakraborty, S., Hur, C., Lahav, O., Vafeiadis, V.: Promising 2.0: global optimizations in relaxed memory concurrency. In: Donaldson, A.F., Torlak, E. (eds.) Proceedings of the 41st ACM SIGPLAN International Conference on Programming Language Design and Implementation, PLDI 2020, London, UK, June 15-20, 2020. pp. 362–376. ACM (2020)

[23] Manson, J., Pugh, W., Adve, S.V.: The java memory model. In: Palsberg, J., Abadi, M. (eds.) Proceedings of the 32nd ACM SIGPLAN-SIGACT Symposium on Principles of Programming Languages, POPL 2005, Long Beach, California, USA, January 12-14, 2005. pp. 378–391. ACM (2005)

[24] Musuvathi, M., Qadeer, S.: Iterative context bounding for systematic testing of multithreaded programs. In: Ferrante, J., McKinley, K.S. (eds.) Proceedings of the ACM SIGPLAN 2007 Conference on Programming Language Design and Implementation, San Diego, California, USA, June 10-13, 2007. pp. 446–455. ACM (2007)

[25] Norris, B., Demsky, B.: Cdschecker: checking concurrent data structures written with C/C++ atomics. In: Hosking, A.L., Eugster, P.T., Lopes, C.V. (eds.) Proceedings of the 2013 ACM SIGPLAN International Conference on Object Oriented Programming Systems Languages & Applications, OOPSLA 2013, part of SPLASH 2013, Indianapolis, IN, USA, October 26-31, 2013. pp. 131–150. ACM (2013)

[26] Norris, B., Demsky, B.: A practical approach for model checking C/C++11 code. ACM Trans. Program. Lang. Syst. **38**(3), 10:1–10:51 (2016)

[27] Paviotti, M., Cooksey, S., Paradis, A., Wright, D., Owens, S., Batty, M.: Modular relaxed dependencies in weak memory concurrency. In: Müller, P. (ed.) Programming Languages and Systems - 29th European Symposium on Programming, ESOP 2020, Held as Part of the European Joint Conferences on Theory and Practice of Software, ETAPS 2020, Dublin, Ireland, April 25-30, 2020, Proceedings. Lecture Notes in Computer Science, vol. 12075, pp. 599–625. Springer (2020)

[28] Post, E.L.: A variant of a recursively unsolvable problem. Bull. Amer. Math. Soc. **52**, 264–268 (1946)

[29] Qadeer, S., Rehof, J.: Context-bounded model checking of concurrent software. In: Halbwachs, N., Zuck, L.D. (eds.) Tools and Algorithms for the Construction and Analysis of Systems, 11th International Conference, TACAS 2005, Held as Part of the Joint European Conferences on Theory and Practice of Software, ETAPS 2005, Edinburgh, UK, April 4-8, 2005, Proceedings. Lecture Notes in Computer Science, vol. 3440, pp. 93–107. Springer (2005)

[30] Svendsen, K., Pichon-Pharabod, J., Doko, M., Lahav, O., Vafeiadis, V.: A separation logic for a promising semantics. In: Ahmed, A. (ed.) Programming Languages and Systems - 27th European Symposium on Programming, ESOP 2018, Held as Part of the European Joint Conferences on Theory and Practice of Software, ETAPS 2018, Thessaloniki, Greece, April 14-20, 2018, Proceedings. Lecture Notes in Computer Science, vol. 10801, pp. 357–384. Springer (2018)

[31] Tomasco, E., Nguyen, T.L., Fischer, B., Torre, S.L., Parlato, G.: Using shared memory abstractions to design eager sequentializations for weak memory models. In: Cimatti, A., Sirjani, M. (eds.) Software Engineering and Formal Methods - 15th International Conference, SEFM 2017, Trento, Italy, September 4-8, 2017, Proceedings. Lecture Notes in Computer Science, vol. 10469, pp. 185–202. Springer (2017)

[32] Torre, S.L., Madhusudan, P., Parlato, G.: Context-bounded analysis of concurrent queue systems. In: Ramakrishnan, C.R., Rehof, J. (eds.) Tools and Algorithms for the Construction and Analysis of Systems, 14th International Conference, TACAS 2008, Held as Part of the Joint European Conferences on Theory and Practice of Software, ETAPS 2008, Budapest, Hungary, March 29-April 6, 2008. Proceedings. Lecture Notes in Computer Science, vol. 4963, pp. 299–314. Springer (2008)

[33] Torre, S.L., Madhusudan, P., Parlato, G.: Reducing context-bounded concurrent reachability to sequential reachability. In: Bouajjani, A., Maler, O. (eds.) Computer Aided Verification, 21st International Conference, CAV 2009, Grenoble, France, June 26 - July 2, 2009. Proceedings. Lecture Notes in Computer Science, vol. 5643, pp. 477–492. Springer (2009)

[34] Torre, S.L., Madhusudan, P., Parlato, G.: Model-checking parameterized concurrent programs using linear interfaces. In: Touili, T., Cook, B., Jackson, P.B. (eds.) Computer Aided Verification, 22nd International Conference, CAV 2010, Edinburgh, UK, July 15-19, 2010. Proceedings. Lecture Notes in Computer Science, vol. 6174, pp. 629–644. Springer (2010)

